# Gender gaps in Mathematics and Language: The bias of competitive achievement tests

**DOI:** 10.1371/journal.pone.0283384

**Published:** 2023-03-22

**Authors:** Oscar Arias, Catalina Canals, Alejandra Mizala, Francisco Meneses

**Affiliations:** 1 Institute of Education, Center for Advanced Research in Education, Universidad de Chile, Santiago, Chile; 2 School of Public Affairs, Arizona State University, Phoenix, Arizona, United States of America; 3 Department Industrial Engineering, Institute of Education and Center of Applied Economics, Center for Advanced Research in Education, Universidad de Chile, Santiago, Chile; 4 Millennium Nucleus on Intergenerational Mobility: From Modeling to Policy (MMOVI), Santiago, Chile; University of Gottingen, GERMANY

## Abstract

This research paper examines the extent to which high-stakes competitive tests affect gender gaps in standardized tests of Mathematics and Language. To this end, we estimate models that predict students’ results in two national standardized tests: a test that does not affect students’ educational trajectory, and a second test that determines access to the most selective universities in Chile. We used data from different gender twins who took these tests. This strategy allows us to control, through household fixed effects, the observed and unobserved household characteristics. Our results show that competitive tests negatively affect women. In Mathematics, according to both tests, there is a gender gap in favor of men, which increases in the university entrance exam, especially for high-performance students. As the literature review shows, women are negatively stereotyped in Mathematics, so this stereotype threat could penalize high-achieving women, that is, those that go against the stereotype. In Language tests, women outperform men in the standardized test taken in high school, but the situation is reversed in the university entrance exam. From our analysis of Chilean national data, we find no evidence that the gender effect observed in the competitive test depends on the students’ achievement level. Following the literature, this gender gap may be linked to women’s risk aversion, lower self-confidence, lower preference for competition, as well as the effect of answering a test under time pressure.

## Introduction

Worldwide, women are underrepresented in Science, Technology, Engineering, and Mathematics (STEM) fields, while they are overrepresented in humanities, social sciences, health, and education [1–3]. The over-representation of one gender over the other across fields of study helps explain gender segregation in the labor market [4].

The difference in gender representation across knowledge fields is partly explained by different preferences, where social stereotypes play an important role [3,5–7]. Gender inequalities observed in the fields of study and later in the labor market are also rooted in achievement gender gaps [8,9]. The results of the 2018 Programme for International Student Assessment (PISA) test show that in all participating countries, women outperform men in reading tests, while in many countries, men outperform women in Mathematics [10].

Considering that academic achievement tests are generally used to determine access to different university careers, it is essential to know to what extent high-stakes competitive tests affect gender gaps in academic performance. We hypothesize that competitive testing might increase these gaps due to gender differences in attitudes toward competition, risk aversion, self-confidence, and performance under time pressure [11–21]. In addition, taking into account the existence of stereotypes linking women to underperformance in Mathematics, the competitive tests might be underestimating women’s real cognitive abilities by stereotyping them negatively [22,23].

Within this framework, this research analyzes to what extent high-stakes competitive tests increases gender gaps in Mathematics and Language achievement in Chile. This is relevant as Chilean men have higher numeracy and literacy skills than women, with gender gaps only surpassed by Turkey among the Organization for Economic Co-operation and Development (OECD) countries [24]. We analyze two nationwide tests in order to compare the differences in the gender gap using a competitive and a noncompetitive context. First, we use the information from the 10^th^ grade Educational Quality Measurement System test (*Sistema de Medición de la Calidad de la Educación*, SIMCE for its Spanish acronym), a test that attempts to measure the schools’ performance without reporting the students’ individual results. Second, we analyze the national entrance test to Chilean universities (Prueba de Selección Universitaria, PSU for its Spanish acronym). We rely on data from different gender dizygotic (fraternal) twins who took both tests, which allows us to control for household characteristics that could affect students’ performance.

Previous research has found evidence that competitive testing negatively affects women during high school [25–28] and even in some specific subjects or programs during college [29–31]. Although there is prior evidence in this field, none of the previous studies analyze students’ performance in competitive and noncompetitive standardized national tests together. Furthermore, it is important to determine how general these results are; for example, is the negative effect of competitive tests on women observed in students with different levels of school performance? Is it observed in different subjects? Is it observed in subjects where the gender gap in noncompetitive tests favors women? This research attempts to examine this gap based on a unique data set comprising six cohorts of Chilean different gender twins who have taken competitive and noncompetitive standardized national tests in Mathematics and Language. The wealth of available data allowed us to use an empirical strategy that compares twins who live in the same household and belong to the same cohort but of a different gender. It is worth noting that, in our data, while men outperform women in the noncompetitive Mathematics test, the opposite is true in the noncompetitive Language test; this allows us to explore the effect of competitive tests in subjects where the gender gap has different signs.

Our results show that high-stakes competitive tests negatively affect women’s academic performance. In Mathematics, the gender gap favoring men in the noncompetitive 10^th^ grade SIMCE test increases in the competitive university entrance exam, especially among the highest performing students. In Language, the gender gap in favor of women found in the 10^th^ grade noncompetitive SIMCE test is reversed in the competitive PSU test, a gap that does not depend on the students’ level of academic performance.

The paper is organized as follows. The next section reviews the literature on gender gaps and competitive testing. Following, we describe the Chilean competitive and noncompetitive standardized tests and then, our empirical strategy and the data used. Next, we present and discuss the results and, finally the conclusions.

## Literature review

The evidence shows that gender gaps in standardized Mathematics and Language tests are widespread. While women outperform men in reading when taking the PISA test, in many countries, including Chile, men outperform women in Mathematics [10].

The gender gaps we find in noncompetitive tests like PISA could be exacerbated in high-stakes competitive tests, which is the case of university entrance exams. Previous research has addressed the role played by competitive tests in achievement gender gaps. Azmat et al. [25] study gender differences in several Arts and Science subjects at the high school level. They found that women outperformed men in both low- and high-stakes tests, but the magnitude was higher in low-stakes tests. Montolio and Taberner [30] and Ballen et al. [29] show that men outperform women in high-stakes tests in specific university courses, but the gender gap decreases or is even reversed when the pressure decreases. Similarly, Örs et al. [31]–based on information from applicants for a master’s degree–and Jurajda & Münich [27]–based on data from high school students–conclude that women underperform in high-stakes tests relative to men with similar abilities. Cai et al. [26] and Saygin [28]–both comparing university entrance exam test scores with a noncompetitive evaluation (a mock of the university entrance exam and the school grades, respectively)–find the same result. Moreover, the two studies show a heterogeneous effect, which is stronger in higher-skilled students.

Based on previous research, we review five mechanisms by which competitive tests may affect gender gaps. These are gender gaps in attitudes toward competition, risk aversion, self-confidence, performance in time-constrained settings, and “stereotype threat.” Each is examined separately.

### Gender gaps in attitudes toward competition

Evidence shows that women have a lower preference for competing with others than men [32–36]. However, gender differences in willingness to compete vary across cultures [17,37–40]. Gender gaps in attitudes toward competition also vary according to the task type [41], the performance level of participants [42], and the institutional design linked to measurements [43,44]. A low preference to compete is observed in women from kindergarten and prevails in adolescence and adulthood, although the gap disappears in older populations [34,45]. In addition, it has been found that women are less willing to compete in Mathematics tests, whereas there is no difference in Language tests [46].

Researchers have explored the extent to which competitive environments affect the performance of men and women, but the evidence is inconclusive. Some studies find that in certain tasks, there are no gender gaps, while in other tasks there are differences that favor women [34,39]. A number of studies found no gender differences in competitive task performance [44,47,48], whereas others found performance differences against women in competitive situations [15,16].

For the specific case of competitive tests, Niederle and Vesterlund [14] state that the gap at the top of the Mathematics test score distribution does not necessarily imply a gap in underlying Mathematics abilities. The authors argue that competition pressure–in the case of tests for this subject–could generate gender gaps that overstate differences in abilities. Similarly, Stenlund et al. [49] and Ballen et al. [29] show that high stake tests produce greater anxiety in women compared to men, which could lead to different levels of achievement in competitive tasks.

### Gender gaps in risk aversion

Competition generally implies the risk of losing even when the consequences of it can vary widely. Previous studies have found gender gaps in preference and risk tolerance [11,50], which may lead to such gaps in competitive environments. Most research shows that women have a higher risk aversion than men [11,33,35,39,50–58], but this effect is moderated by culture [17,18,54,57,59]. Women in single-gender contexts are more likely to prefer risky options, indicating that risk preferences are socially learned [60,61].

Some studies suggest that women’s lower willingness to compete, when compared to men, is linked to risk aversion [11,19,34,35,43]. For example, Niederle & Yestrumskas [43] and Croson & Gneezy [11] conclude that women tend to have higher risk aversion and so lower preference for competition than men.

### Gender gaps in self-confidence

Niederle and Verterlund [13] found gender gaps in the willingness to compete, but that risk aversion explained little of it. Rather, they found that the gap was driven by men being more confident and having a greater preference for competing. Di Tommaso et al. [62] show that women have lower self-confidence in their skills, demonstrated by the perception of gender gaps in performance being greater when compared to actual performance.

Further, the self-confidence gender gaps are likely related to gender stereotyping. Niederle and Vesterlund [14] argue that the self-confidence gender gaps play a substantial role in Mathematics–particularly at the right tail of the performance distribution. They show that when comparing scores for the same math test, women show less faith in their abilities due to extensive gender stereotyping in Mathematics (produced and reproduced by interaction with parents and teachers). Preckel et al. [63] find that gender differences in self-confidence are exacerbated in gifted children. The authors state that this might be due to gifted women being more aware of their aptitudes and social expectations about how such aptitudes are used. In other words, as they are more likely to challenge stereotypes by being high achievers in a masculinized field, they are more likely to be exposed to stereotypes. Thus, the authors argue that students might tend to develop and use these specific skills–and achieve success- where domain and gender social expectations match, which is not the case for women in the STEM field.

Gender gaps in self-confidence can affect performance in competitive contexts through “test anxiety.” This has been described as an emotional condition characterized by tension, worrying thoughts, and negative physiological reactions, which result in gender performance gaps [64]. This condition is strongly and negatively related to self-efficacy and self-esteem and is more pronounced in high-stakes test contexts and perceived test difficulty [64]. It predominantly affects women [49,65,66], is strongly related to mathematics anxiety [67], and develops early in school [68].

### Gender gaps in performance in time-constrained settings

Although few studies have compared gender performance under time constraints [69], there is some evidence that gender differences are found in this context. De Paola & Gioia [21] state that women–unlike men–underperform in tests that measure verbal abilities and Mathematics under time pressure. Shurchkov [20] finds that women underperform men in Mathematics under time pressure. For verbal tests, women are more willing to compete and their results are higher, even outperforming men, in a low-pressure context. Dilmaghani’s [69] analysis of time trial chess competition–where players have a time limit for each move–shows that women underperform men with similar ranking. Again, this effect is greater for women with a higher skill ranking. Finally, Voyer’s [70] meta-analysis of studies comparing gender gaps in spatial ability tests–imaginary object rotation—concludes that gaps are greater in time trial tests, with a linear relationship between the performance gender gap and the task deadline.

### Stereotype threat

A “stereotype threat” is when someone is at risk of confirming a negative stereotype associated to their group as a personal characteristic [71]. For example, when a group is stereotyped as low-performing, their actual cognitive abilities could be underestimated by test scores [22,23,71]. The stereotype threat affects such populations negatively in at least three ways: it produces a physiological response to stress, triggers a tendency to actively monitor performance, and generates self-effort to suppress negative thoughts or emotions. In negatively stereotyped groups, these effects could underestimate actual cognitive ability, measured by test scores, due to working memory overload as a result of stress [23,72].

Gender stereotyping linked to Mathematics has been found during the first years of school education [73] and experienced by pedagogy students [74] and teachers [75]. Therefore, the stereotype threat could negatively affect women’s performance in high-stakes Mathematics tests. In addition, women have lower self-confidence in their Mathematics knowledge and greater anxiety in this field [67,76], which is probably also related to gender stereotypes.

Research shows that in stereotyped environments, women’s performance in Mathematics is affected by the stereotype threat [77]. Bedyńska et al. [78] find similar results with chronic exposure to stereotype threat; In Mathematics, this stereotype affects the performance of women who are strongly identified with their gender (i.e., consider gender to be an important part of their social identity). Nosek et al. [79] analyzed the Trends in International and Science Study (TIMSS), an international noncompetitive test. They found that national stereotypes about the relationship between gender and science partially explain the gap in Mathematics and Science test scores. Similarly, Nollenberger et al. [80] show that cultural beliefs about gender roles explain most of the gender gap in Mathematics among second-generation immigrants. However, in a large-scale experiment in high schools in the Netherlands, Flore et al. [81] found no evidence of stereotype threat affecting women’s performance in Mathematics.

Even though stereotype threat could also affect results in Language, where men tend to underperform, this has not been studied as intensively as in Mathematics [82], and results have not been conclusive. Pansu et al. [83] show that men underperform compared to women in reading tests in a context with high stereotype threat, whereas they outperform women in contexts with low stereotype threat. However, Bedyńska et al. [82] present mixed results, and Chaffe [84] finds little or no effect in an experimental analysis of stereotype threat in Language. In short, the evidence on the stereotyped threat is inconclusive, and further research on its role in competitive contexts is necessary.

## Performance tests in Chile

During primary and secondary education in Chile, students are tested for their academic achievement. School grades are expressed with a 1.0 to 7.0 numeric scale, where 4 is the minimum passing grade [85]. However, school grades cannot be directly compared across schools as schools are not equally demanding of their students, leading to different distributions of school grades across schools [86].

The SIMCE test was created to compare the results of schools nationwide and is a standardized test taken by 4^th^, 8^th^, and 10^th^ grade students. The SIMCE test annually assesses Mathematics and Language skills and knowledge based on the national curriculum [87]. There are also SIMCE tests for the natural and social sciences, English, computer science, and technology (ICTs), although these tests are not administered every year. The 10^th^ grade SIMCE test consists of approximately 45 questions, lasts 90 minutes, and as it is not a student´s achievement test, examiners have the discretion to extend the time period if a student requires it [88]. The SIMCE test is a noncompetitive test, the results have no impact on the qualification or promotion of students. Moreover, students, parents, teachers, and principals cannot access individual student scores, only aggregated scores at the school level.

The Chilean post-secondary system is composed of universities and vocational institutions. There are two types of universities: traditional universities (27 public and private institutions created before the 1980 higher education reform) and non-traditional universities (more than 30 private universities created after 1980). Chile has a single centralized admission system for its traditional universities (*Sistema Único de Admisión*, SUA, by its Spanish acronym), administered by the Department of Educational Evaluation, Measurement and Registration (*Departamento de Evaluación*, *Medición y Registro Educacional*, DEMRE, by its Spanish acronym) based at the University of Chile. Some private non-traditional universities have increasingly joined SUA as Chile´s most prestigious and selective universities are part of the system.

The SUA university entrance selection process is based on the students’ high school grades and their scores in the national university entrance test (PSU). PSU scores are fitted to a normal distribution with a range between 150 to 850, and a mean and median of 500 points. To participate in this selection process, students are required to take the Mathematics and Language PSU tests. They may take the optional social sciences or natural sciences tests, which are required for certain careers. Mathematics and Language PSU tests last 150 minutes, consist of 80 close-ended questions, and have the purpose of measuring students’ knowledge and skills according to the national curriculum [89]. Students apply through a website, ranking their ten preferred programs from most to least preferred. A program corresponds to a university-career binomial; for each program, students have an application score, calculated by applying weights to students’ PSU scores and high school grade point averages, previously rescaled to a range of 150 to 850, consistent with PSU tests. Once students apply, university programs fill their openings in strict order based on the students’ application scores. Unlike SIMCE, the PSU test is a competitive test, and the most prestigious universities and careers seek students with the highest scores. Students compete for the best possible score because it will determine their admission to the university and the career of their interest. Also, the obtained score could also affect their chances of obtaining a scholarship.

Since the PSU is a competitive high-stakes test taken under time constraints, we expect the five mechanisms mentioned in the literature review to affect gender gaps. In contrast, the SIMCE test is noncompetitive and has no direct consequences for the students; therefore, gender gaps based on attitudes toward competition and risk are unlikely to play a role. Nonetheless, gender gaps in self-confidence and the stereotype threat -particularly in the case of women’s performance in Mathematics- might affect their performance in the SIMCE test. However, as the test has no consequences in the future, the anxiety associated with the test is expected to be lower. As mentioned, SIMCE is a noncompetitive test where examiners are allowed to give students more time to take the test if needed, so it is unlikely that time-constrains influence the results.

Given the above and the evidence reviewed, we expect that:

- Gender gaps in attitudes toward competition, risk aversion, and performance in time-constrained environments negatively affect women’s performance in the PSU but not in the SIMCE tests.
- Gender gaps in self-confidence would negatively affect women’ performance in both tests, but the effect would be greater in the PSU test, since this test is associated with greater test anxiety. Furthermore, if gender gaps in self-confidence are larger among gifted students, the achievement gap would be even larger for high achieving women.
- In the case of Mathematics, stereotype threat would negatively affect the performance of women with high performance in both the SIMCE and PSU tests. However, the effect is expected to be greater in the PSU test, due to the higher anxiety associated with a high-stakes test. If there is a negative stereotype about men’s performance in Language, then stereotype threat would also affect high-achieving men more.

Therefore, if we find that women are negatively affected by the PSU test, compared to the SIMCE test, this result would be consistent with three mechanisms: gender gaps in attitudes toward competition, risk aversion, self-confidence, and performance in time-constrained environments. However, our analysis will not allow us to distinguish which of the three mechanisms is operating. If we find that high achieving women underperform in the Mathematics PSU test, it would be consistent with both a stereotype threat and greater gender gaps in self-confidence of gifted students. Also, if high-achieving men underperform in the PSU Language test, it would suggest a stereotype threat effect. However, our design cannot directly test for these mechanisms.

## Methodology and data

In this study, we analyze data from the cohorts of students who applied to enter Chilean universities between the years 2015 and 2020. All the data used is secondary and anonymized; they come from administrative data and questionnaires (SIMCE and PSU) that are answered at the time of carrying out the tests. At the beginning of these questionnaires, those who respond are informed that the information may be used anonymously by the Ministry of Education, DEMRE, and other institutions that carry out research in education. The questionnaire, in the case of SIMCE, is voluntary; however, it has a high response rate.

Since the main objective is to compare gender differences between competitive and noncompetitive tests, we use data from both types of tests for Mathematics and Language. First, we used the PSU (competitive test) scores data provided by DEMRE and the Ministry of Education. The data is publicly available. To access the data requires completing an online form on DEMRE webpage (available at https://ayuda.demre.cl/, selecting the issue: *Otras consultas*, “Other inquiries" in Spanish). We asked DEMRE to include a students’ unique identifier called “MRUN", in the data provided, which allowed us to merge this data with that from other sources. Once DEMRE responds to the researchers’ request, this response letter should be sent to the Ministry of Education to obtain the information (estadisticas@mineduc.cl).

We also used the SIMCE test (noncompetitive) data. The cohorts we analyzed took the SIMCE test in 4^th^ grade, as well as in 10^th^ grade. The SIMCE scores were provided by the *Agencia de Calidad de la Educación* (Education Quality Agency). In Chile, when students take the SIMCE test, questionnaires are administered to teachers, parents, and students to provide a characterization of the students’ socioeconomic status and students’ expectations about their university entrance. The SIMCE test data and the questionnaires answered by parents, students, and teachers at the time of the test are publicly available upon request. The *Agencia de Calidad de la Educación* has a webpage with an application form and instructions for such a request (https://www.agenciaeducacion.cl/simce/). We also asked them to include the student’s unique identifier (“MRUN").

The models were estimated to predict the scores of the students in both tests, which were previously standardized to a normal distribution with a mean equal to zero and a standard deviation equal to one. The score in each subject of a given test was standardized by cohort. Our estimates are controlled for students’ prior academic performance, as measured by SIMCE test scores taken in 4^th^ grade and their Math and Language scores from the year prior to the year they took the test. (9^th^ grade for the case of the 10^th^ grade SIMCE test, and 12^th^ grade for the case of the PSU test). School grades were standardized by school. To do this, we take a student’s school grades, subtract the average school grades of the students in their cohort at his/her school, and divide by the standard deviation of the school grades of the same students. This was done since the distribution of school grades can vary between schools, because schools do not grade consistently.

We also used students’ school attendance data from the year prior to taking 10^th^ grade SIMCE test and PSU test (9^th^ and 12^th^ grade, respectively) as a control variable. School attendance rate is measured as the percentage of attended days.

The information about school grades and the school attendance rate of the students was provided by the Ministry of Education. School attendance data with students´ identifier (MRUN) can be directly downloaded from the Ministry of Education webpage (https://datosabiertos.mineduc.cl/asistencia-declarada-mensual-2/). School grade data by subject is in a dataset called *Notas por estudiante y subsector* (Grades by Student and Subsector). The dataset with the same identifier is also publicly available, but it must be requested by an e-mail to the Ministry of Education (estadisticas@mineduc.cl).

Table 1 shows the years that each cohort attended 4th, 9th, 10^th^, and 12^th^ grade and the corresponding year of admission to university. These are the years for which we have the information used in this paper.

**Table 1:** Cohorts of students analyzed.

Cohort	4^th^ grade	9^th^ grade	10^th^ grade	12^th^ grade	University admission process
**2015**	2006	2011	2012	2014	2015
**2016**	2007	2012	2013	2015	2016
**2017**	2008	2013	2014	2016	2017
**2018**	2009	2014	2015	2017	2018
**2019**	2010	2015	2016	2018	2019
**2020**	2011	2016	2017	2019	2020

To estimate the model, we rely on dizygotic twins that include at least one boy and one girl; for the sake of simplicity we speak about twin couples, but it should be noted that we also consider triplet or quadruplets that include at least one boy and one girl. Dizygotic twins do not share the same genetic content (their resemblance is the same as the one that two siblings born in different moments could have). The fact that we have pairs of twins who are born at the same time and in the same household allows us to control for both observed variables (such as household income and parents’ educational level), and for unobserved variables, linked to the household, that could be correlated with school performance (such as parenting styles and the amount and quality of time spent parenting and supporting children at school). As explained, we use a unique identifier for each pair of twins provided by the Ministry of Education. In our PSU data request to DEMRE and the Ministry of Education, we asked them to use the students’ personal data, which is not publicly available, in order to create this unique identifier by household and include it in the PSU dataset. Therefore, we can include household fixed effects in the models, allowing us to control for observed and unobserved household characteristics that are constant within a given household.

Table 2 shows the number of twins per cohort–notice that there could be an odd number when there are triplets. Although the estimations presented in the main test only include different gender twins, additional estimations considering the total population are provided in S1 Appendix.

**Table 2:** Number of twins per cohort.

Cohort	Cases
**2015**	377
**2016**	367
**2017**	342
**2018**	304
**2019**	375
**2020**	381
**Total**	2146

Table 3 presents the descriptive statistics of the 2015–2020 cohorts of the student population that took the PSU and 10^th^ grade SIMCE tests. We include the total population (part A) and the twin subpopulation (part B). Interestingly when compared to the rest of the population, twins have higher academic achievement–measured by SIMCE and PSU tests-, higher high-school attendance, higher expectations of entering the university, and tend to study in schools with better SIMCE test results. Considering that the socioeconomic composition of schools is positively correlated with their average test scores [90], the differences between the total population and the twin population are possibly explained by the higher socioeconomic status of households with twin children. This difference could be related to the fact that the birth of twins is associated with the use of assisted reproductive treatments, which can be expensive, and that multiple pregnancies represent greater care and costs, so that households of high income can bring these pregnancies to a successful end [91,92].

**Table 3:** Descriptive statistics of the student population, total population and twins population, 2015–2020 cohorts.

	Total population					Twin population				
Variable	N	Mean	Standard Deviation	Min	Max	N	Mean	Standard deviation	Min	Max
**Women**	880719	0.53	0.5	0	1	1836	0.5	0.5	0	1
**Household-income (thousands of CLP 2020)**	696177	667.59	645.44	50	2628.9	1614	1046.74	861.35	50	2628.9
**Parents’ educational level (average years of schooling)**	707169	12.07	3.20	0.00	21.00	1497	13.37	3.43	0.00	21.00
**4^th^ grade Mathematics SIMCE score1**	703477	0.37	0.91	-3.06	2.51	1664	0.51	0.88	-2.43	2.51
**4^th^ grade language SIMCE score^1^**	703115	0.34	0.9	-3.29	2.33	1665	0.45	0.87	-2.7	2.26
**9^th^ grade Mathematics grades^2^**	847993	0.22	0.95	-4	4.28	1815	0.34	0.96	-3.02	3.41
**9^th^ grade language grades^1^**	841782	0.22	0.94	-4.85	4.17	1792	0.32	0.95	-3.24	3.26
**9^th^ grade Attendance rate (%)**	849045	92.94	7.72	0	100	1815	94.27	6.02	33	100
**10^th^ grade Mathematics SIMCE score^1^**	857353	0.22	0.96	-2.73	2.52	1787	0.56	0.92	-2.46	2.52
**10^th^ grade language SIMCE score^1^**	848359	0.19	0.97	-2.63	2.98	1789	0.43	0.95	-2.2	2.83
**12^th^ grade Mathematics grades^2^**	877073	0.11	0.97	-5.75	4.88	1810	0.23	0.94	-2.73	2.86
**12^th^ grade language grades^2^**	852046	0.11	0.96	-9.05	4.33	1743	0.19	0.96	-3.04	3.41
**12^th^ grade attendance rate (%)**	879481	91.39	8.03	0	100	1832	92.46	7.12	0	100
**Students with university entrance expectation^3^**	801407	0.77	0.42	0	1	1674	0.86	0.35	0	1
**Previous 10^th^ grade Mathematics SIMCE score of the school^1^**	863217	0.26	0.99	-2.77	2.85	1782	0.66	1.02	-2.03	2.62
**Previous 10^th^ grade language SIMCE score of the school^1^**	863072	0.25	0.98	-2.96	3.25	1780	0.59	0.99	-2.16	3.08
**10^th^ grade Mathematics SIMCE score of the school^1^**	880729	0.26	0.99	-2.84	2.99	1836	0.64	1.02	-1.81	2.59
**10^th^ grade language SIMCE score of the school^1^**	880661	0.25	0.98	-2.95	3.27	1836	0.58	0.99	-2.33	2.68
**Mathematics PSU^1^**	869603	0.06	0.99	-3.18	3.18	1817	0.45	1.06	-2.64	3.18
**Language PSU^1^**	879155	0	0.98	-3.18	3.18	1836	0.32	1	-3.18	3.18

^1^ SIMCE and PSU variables were standardized to a normal distribution with mean equal to zero, and standard deviation equal to 1—standardization made by cohort.

^2^ School grades were standardized to a normal distribution with mean equal to zero, and standard deviation equal to 1—standardization made by cohort and school.

^3^ Expectations of entering the university is a dummy variable. Its value equals 1 when the student in 10^th^ grade expects to attend the university, and 0 otherwise.

As mentioned above, we estimate and compare models to predict the scores of the PSU and 10^th^ grade SIMCE tests. The score of one of the twins *i*, in 10^th^ grade SIMCE or PSU test *T* in the subject of Mathematics or Language *A* is given by the Eq (1), where *woman* is a dummy variable indicating whether the student is a woman, *Ach*_*i*,*A*_ is a vector of variables associated to the student’s academic achievement, *Sch*_*i*,*A*_ is the school’s 10^th^ grade SIMCE score in the *A* subject, *exp* is a dummy variable indicating the university entrance expectation of the student in 10^th^ grade, *α* is a constant, *β*_1_−*β*_4_ are parameter vectors, *α*_*H*_ is a household fixed effect (twin couples), *ε*_*i*_ is a random error. Note that in the estimations to predict PSU score, *Sch*_*i*,*A*_ corresponds to the school average SIMCE score of the 10^th^ grade test in the year in which the student attended the 10^th^ grade, while in the estimation to predict 10^th^ grade SIMCE score, it corresponds to the school’s average score on this test the year before the student attended that grade. By including the household fixed effect, we are able to control for characteristics that are the same across siblings. Thus, we are comparing gender gaps between brother and sister of the same twin couple.


$${Score}_{i,T,A}=\alpha +\beta_{1}{woman}_{i}+\beta_{2}{Ach}_{iA}+\beta_{3}{Sch}_{i,A}+\beta_{4}{exp}_{i}+\alpha_{H}+\varepsilon_{i}$$ (1)

Vector *Ach*_*i*,*A*_ includes the following variables:

1. Previous Mathematics and Language SIMCE results: the student’s 10^th^ grade SIMCE score is used in the estimations to predict PSU scores, while student’s 4^th^ grade SIMCE score is used in the estimations to predict 10^th^ grade SIMCE score.
2. Attendance rate (%): 9^th^ grade attendance rate is used to predict the 10^th^ grade SIMCE and 12^th^ grade attendance rate is used to predict PSU test score.
3. School grades of subject *A*: grades obtained in 9^th^ grade are used to predict 10^th^ grade SIMCE scores and grades obtained in 12^th^ grade are used to predict PSU test score.

**Fig 1:**
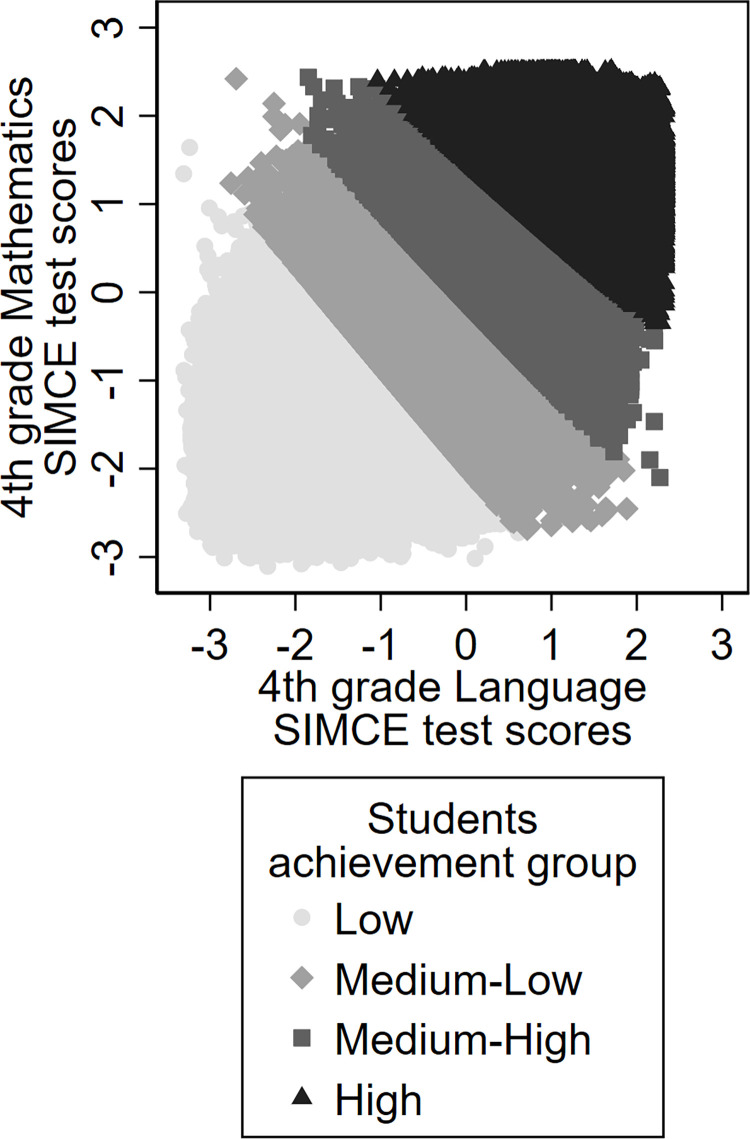
Student’s achievement groups. Each dot represents a student according to their 4^th^ grade Language SIMCE (x-axis) and Mathematics (y-axis) score. Both scores are measured in units of standard deviation. The students were classified into groups according to both scores, using the k-means methodology with Euclidean distance; the dot color shows the group each student is in.

In addition, to analyze whether gender effects vary according to the students’ previous performance, we estimate two variants of this model. The first variant (model 2) uses as predictors the previous SIMCE test in subject A for men and women separately. The second variant (model 3) includes a classification of students into four groups according to their performance in Mathematics and Language in 4^th^ grade; the groups are: (1) low-achieving students, (2) medium-low achieving students, (3) medium-high achieving students and (4) high achieving students. In this model variant, we use the students (men/women) membership in one of these four groups as predictors of test scores. The groups were identified using the k-means methodology with Euclidian distance, an iterative technique that classifies the cases in *k* groups with the aim of minimizing the average distance of the cases from the center of their groups [93], based on the two variables included in the clustering process that is Mathematics and Language 4^th^ grade SIMCE test scores. Fig 1 shows Mathematics and Language performance of each of the four groups identified. S2 Appendix provides the analysis that justifies a 4-group classification. In addition, as a robustness check, S3 Appendix shows estimates using 2, 3, and 5 achievement groups identified with the same methodology, as well as estimates using alternative methods of 3 and 4 achievement groups.

The three variants of the model are estimated by ordinary least squares to predict PSU and 10^th^ grade SIMCE Mathematics and Language scores.

## Results

### Gender gap in Mathematics

Table 4 shows the estimation of the three variants of the model to predict 10^th^ grade Mathematics SIMCE and PSU test results, where the effect of the student’s gender in the competitive test is compared to the noncompetitive test.

**Table 4:** Estimated coefficient. Models to predict Mathematics performance in competitive (PSU) and noncompetitive (10^th^ grade SIMCE) tests.

	10^th^ grade SIMCE			PSU		
	Model 1	Model 2	Model 3	Model 1	Model 2	Model 3
**Women**	-0.075**	-0.059		-0.143***	-0.072	
**Men** * **Previous Mathematics SIMCE score**^**1**^		0.206***			0.441***	
**Women** * **Previous Mathematics SIMCE score**^**1**^		0.176***			0.333***	
**Men** * **Medium-low achievement**			0.351***			0.153
**Men** * **Medium-high achievement**			0.537***			0.396***
**Men** * **High achievement**			0.672***			0.546***
**Women** * **Low achievement**			0.118			-0.117
**Women** * **Medium- low achievement**			0.287***			0.042
**Women** * **Medium- high achievement**			0.417***			0.199
**Women** * **High achievement**			0.567***			0.367***
**Previous Mathematics SIMCE score** ^**1**^	0.191***			0.388***		
**Previous language SIMCE score** ^**1**^	0.038	0.039		0.025	0.027	
**10**^**th**^ **grade Mathematics SIMCE school score**^**1**^	0.533***	0.532***	0.538***	0.189*	0.195*	0.391***
**Mathematics grades** ^**2**^	0.371***	0.371***	0.402***	0.284***	0.280***	0.389***
**Attendance rate (%)**	0.012***	0.013***	0.011***	0.001	0	0.000
**Students with university entrance expectation** ^**3**^	0.08	0.078	0.081	0.081	0.065	0.112
**Constant**	-1.204***	-1.236***	-1.2523***	0.016	0.024	-0.264
**N**	1138	1138	1138	1138	1138	1138
**Adjusted R** ^**2**^	0.713	0.713	0.702	0.676	0.679	0.654

^1^ SIMCE and PSU variables were standardized to a normal distribution with mean equal to zero, and standard deviation equal to 1—standardization made by cohort.

^2^ School grades were standardized to a normal distribution with mean equal to zero, and standard deviation equal to 1—standardization made by cohort and school.

^3^ Expectations of entering university is a dummy variable. Its value equals 1 when the student in 10^th^ grade expects to attend the university, and 0 otherwise.

* p<0.05

** p<0.01

*** p<0.001. Estimations made including household fixed effects.

**Fig 2:**
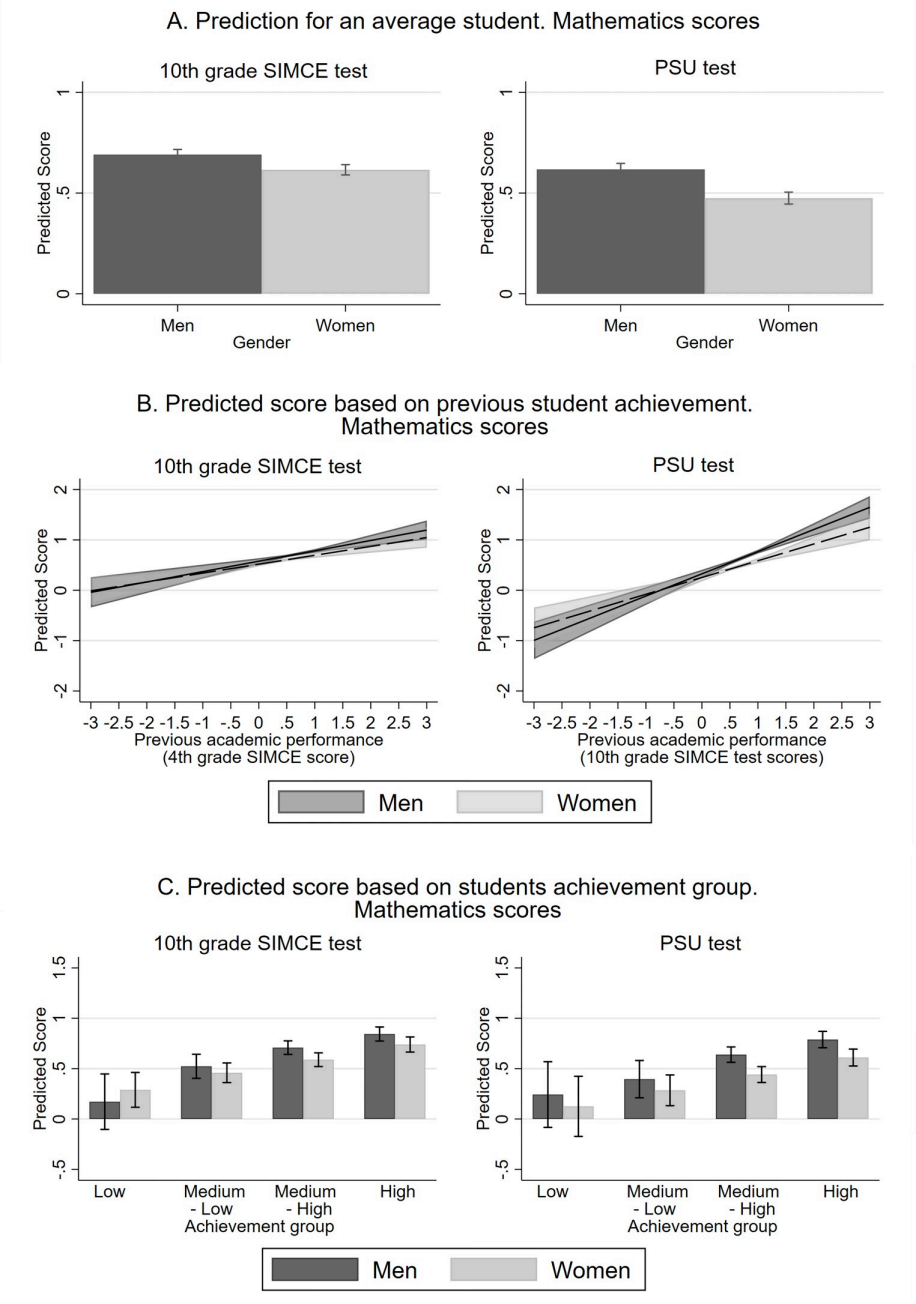
Predictions of Mathematics scores in competitive (PSU) and noncompetitive (10^th^ grade SIMCE) tests. The figure shows predictions for students with average characteristics but different gender (panel A), different previous Mathematics SIMCE test scores and gender (panel B), and different achievement groups and gender (panel C). All the predictions show 95% confidence intervals. The estimations of panels A, B, and C were made with models (1), (2), and (3), respectively. Table 4 shows the coefficients of those models.

Fig 2 shows the predictions of Mathematics scores in competitive (PSU) and noncompetitive (10^th^ grade SIMCE) tests. Panel A shows the predicted scores on both tests estimated using model 1 for twins with average characteristics but different gender. Panel B shows the predicted scores on both tests estimated using model 2, that is, considering students´ previous SIMCE Mathematics test performance separated by gender as an explanatory variable. Panel C shows the predicted scores using model 3, i.e., according to students´ gender and the different achievement groups to which they belong. The predictions show 95% confidence intervals.

Model (1) in Table 4 demonstrates that women’s scores are negatively affected in the competitive test. Even though women tend to underperform men in both tests, the gap has a different magnitude depending on the test. While women obtain scores of 0.075 units of standard deviation less than men in 10^th^ grade SIMCE test, the gap almost doubles in the competitive PSU test (0.143 standard deviations). In Fig 2 Panel A, although the average woman underperforms when compared to men in both tests, the gap between them increases in the competitive PSU test.

To examine the extent to which the gender gap interacts with student academic performance, we analyze the estimates of models (2) and (3). The results demonstrate that high-performing women are the most affected by competitive tests. Specifically, this is shown in the second model found in Table 4, when we introduce the interactions between the gender variable and the previous SIMCE Mathematics test score. As expected, women and men with higher previous scores in the SIMCE test obtain better scores in both tests (PSU and 10^th^ grade SIMCE). Additionally, the coefficients of men have a higher magnitude than those of women in both tests. However, in the case of the 10^th^ grade SIMCE test, the differences are not statistically significant (p-value = 0.395), but they are for the PSU test (p-value = 0.020). Fig 2 panel B shows the differences between these effects graphically by using the predicted test scores for twins with average characteristics, different previous SIMCE test scores, and different gender. In the case of 10^th^ grade SIMCE test, gender gaps are not statistically significant for most previous achievement levels, with the exception of those students with performance levels higher than 0.5 standard deviations. However, the differences may not be statistically significant for students with previous performance greater than 2.5 standard deviations as only a few students achieve such high scores.

The estimates of model 3 allow us to corroborate the relationship between the gender effect in competitive tests and the students’ previous performance. The model (3) in Table 4 incorporates the interactions between the women variable and the performance group of each student in the 4^th^ grade SIMCE test, distinguishing those students with low, medium-low, medium-high, and high achievement. Table 5 presents the coefficients of men and women with the same student achievement group and compares the gap between them. With 95% confidence, we observe that medium- high and high achievement men outperform women in the 10^th^ grade SIMCE and PSU tests; however, the gender gap has a higher magnitude in PSU tests. The same result is confirmed in Fig 2 Panel C, which shows the scores predicted by model 3 for twins with average characteristics, different gender, and previous achievement group. S3 Appendix shows additional estimations for model (3), considering different groups of students according to their performance. All the estimates lead to a robust result, i.e., an increase in the gender gap in competitive tests for high performance women.

**Table 5:** Gender gaps comparison for different levels of previous achievement. Estimations based on Model (3) for Mathematics.

		Coefficient			
	Achievement group	Men	Women	Gap	p-value*
**SIMCE**	**Low**	0.00	0.12	-0.12	0.49
	**Medium-Low**	0.35	0.28	0.07	0.40
	**Medium-High**	0.53	0.42	0.11	0.02
	**High**	0.67	0.57	0.10	0.02
**PSU**	**Low**	0.00	-0.12	0.12	0.60
	**Medium-Low**	0.15	0.04	0.11	0.35
	**Medium-High**	0.40	0.20	0.20	0.00
	**High**	0.55	0.37	0.18	0.00

*p-value in test F in order to prove the null hypothesis of coefficient equality.

In addition, S1 Appendix shows estimates for the three variants of the model, using the total population but with no household fixed effect. These estimates show the same patterns seen in the twin population but with some differences in magnitude.

#### Gender gap in Language

**Fig 3:**
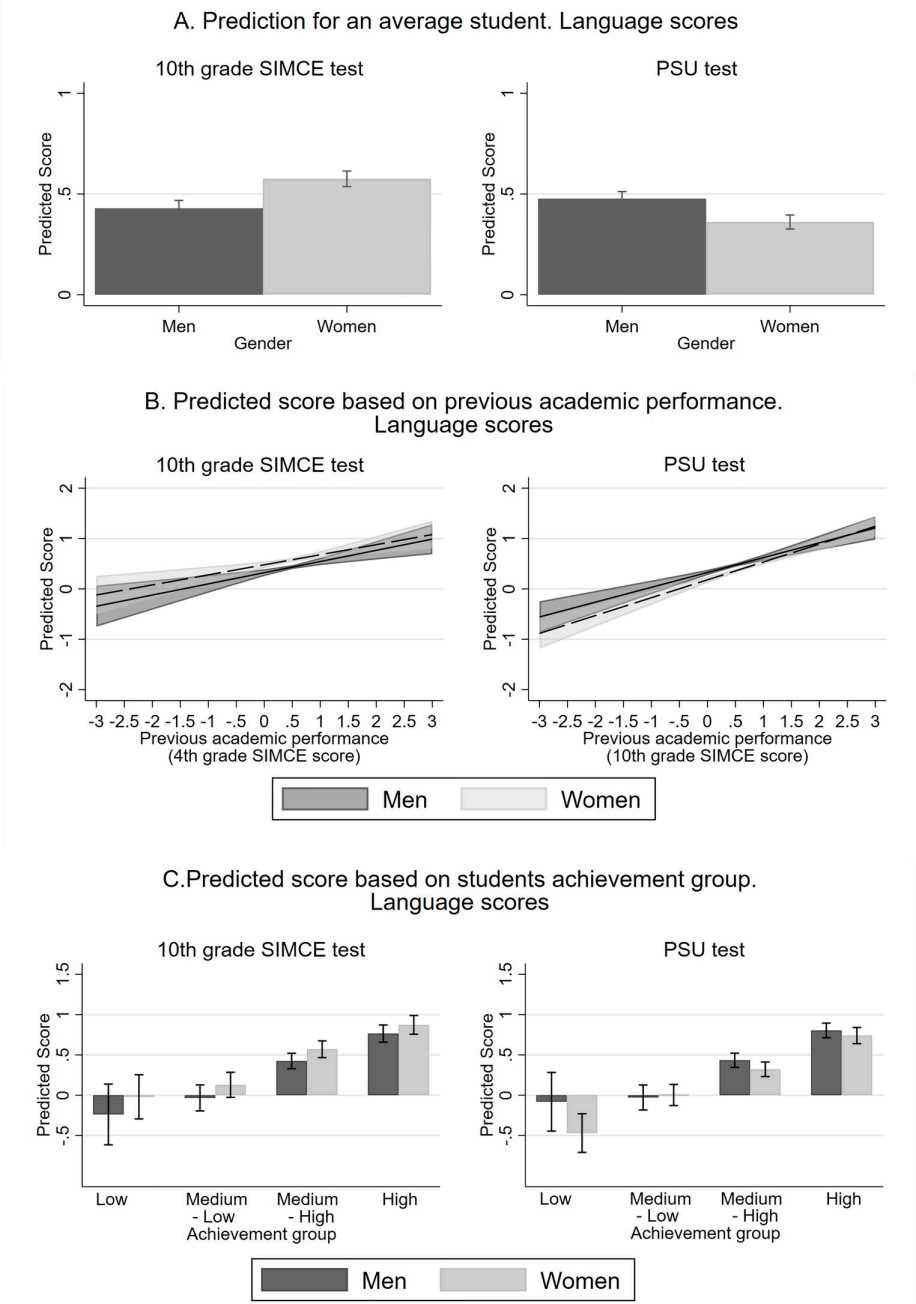
Predictions of Language scores in competitive (PSU) and noncompetitive (10^th^ grade SIMCE) tests. The figure shows predictions for the students with average characteristics and different gender (panel A), different previous Language SIMCE test scores and gender (panel B), and different achievement group and gender (panel C). All the predictions show 95% confidence intervals. The estimations for panels A, B, and C were made with models (1), (2), and (3), respectively. Table 6 shows the coefficients of these models.

Table 6 shows estimations for Language using the same models as those for Mathematics (above). Fig 3 shows the competitive (PSU) and noncompetitive (10^th^ grade SIMCE) test score predictions for Language. Panel A shows the predicted scores on both tests estimated using model (1), for twins with average characteristics but different gender. Panel B shows the predicted scores on both tests estimated using model (2), that is, using the previous performance of students (women/men) in the SIMCE Mathematics test as an explanatory variable. Panel C shows the predicted scores using model (3), i.e., according to the different achievement groups and gender.

**Table 6:** Estimated coefficients. Models to predict the performance in language competitive tests (PSU) and noncompetitive tests (10^th^ grade SIMCE).

	SIMCE 10th grade			PSU		
	Model 1	Model 2	Model 3	Model 1	Model 2	Model 3
**Women**	0.146***	0.157***		-0.116**	-0.146***	
Men * Previous language SIMCE score ^**1**^		0.222***			0.295***	
Women * Previous language SIMCE score ^**1**^		0.200***			0.354***	
Men * Medium-low achievement			0.205			0.053
Men * Medium- high achievement			0.664***			0.515***
Men * High achievement			1.004***			0.885***
Women * Low achievement			0.219			-0.389***
Women * Medium-low achievement			0.368			0.084
Women * Medium-high achievement			0.809***			0.403***
Women * High achievement			1.112***			0.821***
**Previous Mathematics SIMCE score** ^**1**^	0.208***	0.210***		0.241***	0.241***	
**Previous language SIMCE score** ^**1**^	0.211***			0.325***		
**10**^**th**^ **grade Language SIMCE school score**^**2**^	0.202**	0.202**	0.219***	-0.023	-0.02	0.207***
**Language grades**	0.304***	0.304***	0.331***	0.225***	0.224***	0.302***
**Attendance rate (%)**	0.004	0.004	0.003	0	0	0.003
**Students with university entrance expectation** ^**3**^	0.206*	0.205*	0.217***	0.129*	0.135*	0.202***
**Constant**	-0.606	-0.617	-1.008	-0.034	-0.024	-0.791
**N**	1084	1084	1084	1084	1084	1084
**Adjusted R** ^**2**^	0.441	0.440	0.432	0.553	0.555	0.563

^1^ SIMCE and PSU variables were standardized to a normal distribution with mean equal to zero, and standard deviation equal to 1—standardization made by cohort.

^2^ School grades were standardized to a normal distribution with mean equal to zero, and standard deviation equal to 1—standardization made by cohort and school.

^3^ Expectations of entering university is a dummy variable. Its value equals 1 when the student in 10^th^ grade expects to attend the university, and 0 otherwise.

* p<0.05

** p<0.01

*** p<0.001. Estimations made including household fixed effects.

The models show that the negative effect of competitive tests on women is not only in Mathematics, where they tend to obtain lower scores, but are also found in Language, a subject where they outperform men in noncompetitive settings. In particular, the results of model (1) show that the effect of the women variable is statistically significantly in both tests: women have 0.146 standard deviations more than men in the 10^th^ grade Language SIMCE score when controlling for the other variables, but 0.116 standard deviations less than men in the PSU score (see Table 6). Likewise, the predictions for twin couples with average characteristics show that the gender gap in favor of women found in the 10^th^ grade SIMCE test is reversed in the PSU test. (See Fig 3 panel A).

Additionally, Table 6 shows that for Language, unlike Mathematics, there is no evidence that the gender effect in competitive tests is linked to academic performance. In model (2), the coefficient for the women variable is similar to that in model (1). However, for both PSU and 10^th^ grade SIMCE test results, the effect of the previous SIMCE score does not show statistically significant differences between genders (p-value = 0.663 and 0.1416, respectively). Fig 3 panel B shows no large predicted differences for twins with average characteristics, a given score in the previous SIMCE test. This suggests that the effect of gender on competitive Language tests does not depend on prior student performance.

Similarly, the results of model (3) in Table 6 also show that the gender gap in favor of men that we can observe in the PSU test is not generated exclusively by a specific achievement group. Table 7 shows there are significant differences between men and women in two performance groups, when coefficients for the SIMCE and PSU test scores are compared at a given level of academic performance. First, in the low achievement group, there are no statistically significant differences between men and women in the 10^th^ grade SIMCE test, but women outperform men in PSU test. Second, in the medium-high achievement group, there is a gap in favor of women in the 10^th^ grade SIMCE test, which disappears in the PSU test. There are no statistically significant gaps between men and women (with 95% confidence) in the medium-low and high achievement groups. Moreover, Fig 3 panel C shows predicted scores for twins with average characteristics according to gender and achievement group; we do not find statistically significant gender differences. The S3 Appendix shows model estimates using alternative methods to classify students according to their previous performance in the 4^th^ grade SIMCE test. We do not find consistent results in these estimates indicating that the gender gap in favor of men in competitive tests can only be found at a certain level of achievement.

**Table 7:** Gender gaps comparison for different levels of previous achievement. Estimations based on Model (3) for Language.

		Coefficient			
	Achievement group	Men	Women	Gap	p-value*
**SIMCE**	**Low**	0.00	0.22	-0.22	0.29
	**Medium-Low**	0.21	0.37	-0.16	0.11
	**Medium-High**	0.66	0.81	-0.15	0.04
	**High**	1.00	1.11	-0.11	0.14
**PSU**	**Low**	0.00	-0.39	0.39	0.04
	**Medium-Low**	0.05	0.08	-0.03	0.74
	**Medium-High**	0.52	0.40	0.11	0.08
	**High**	0.89	0.82	0.07	0.25

*Value-P in F test in order to prove the null hypothesis of coefficient equality.

Based on the above analysis, we cannot affirm that the interaction between gender and students’ previous scores affects their performance in Language on competitive tests. This would suggest that the shift from a gap that favors women on the 10th grade SIMCE test to one that favors men on the PSU test is a cross-sectional trend that is not generated at specific performance levels, but it does not reach a sufficient magnitude to generate statistically significant differences in all performance groups.

S1 Appendix shows estimates for the three variants of the model, using the total population but without household fixed effects. The estimates corroborate our previous findings; i.e., there is a gender gap in favor of women in the SIMCE test, while the opposite occurs in the PSU test. Given the large number of observations for the total population, gender differences are statistically significant for all levels of performance.

### Are there temporal achievement trends in gender gaps?

As there is a two-year period between the two tests (the 10^th^ grade SIMCE test and the PSU, taken after finishing 12th grade) it is possible that the observed differences result from temporal trends in student performance. In Mathematics, where men outperform women in 10^th^ grade, it could be argued that men have a greater ability to improve their academic performance each year, which could explain the increasing gap in favor of men in the PSU test. This phenomenon is known as the Matthew effect, in which those who initially have a higher performance also have a higher rate of growth of their performance [94]. On the other hand, in Language–where women outperform men in 10^th^ grade–it could be argued that men could have a greater ability to improve their performance each year, which would explain part of the gap in favor of men in the PSU test. This would correspond to a compensatory effect, i.e., it is easier to improve when you have lower scores [94].

**Fig 4:**
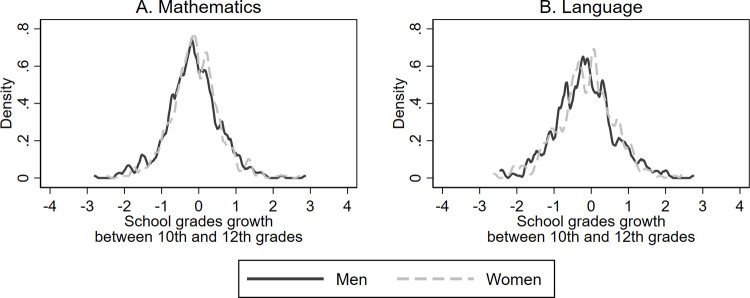
School grades growth between 10^th^ and 12^th^ grade by gender. Panel A shows the growth in Mathematics grades between 10^th^ and 12^th^ grade in men and women. Panel B shows the growth in Language grades between 10^th^ and 12^th^ grade in men and women. The grades are measured in standard deviation units, and the growth is calculated as 12^th^ grade scores minus 10^th^ grade scores.

Although it is unlikely to observe opposite temporary effects in Mathematics and Language–Matthew effect and compensatory effects, respectively- we explore whether the differences found between the 10^th^ grade SIMCE test and the PSU test could be explained by temporal trends in performance improvement. To do this, we analyzed time trends between 10^th^ and 12^th^ grades for men’s and women’s school grades. Fig 4 shows the change in school grades in Mathematics (panel A) and Language (panel B) between grades 10^th^ and 12^th^ for men and women. The grades are measured in standard deviation units, and the growth is calculated as 12^th^ grade scores minus 10^th^ grade scores. We observe that the growth of Mathematics and Language grades is very similar for men and women (value p = 0.52 and value p = 0.41, respectively, using the Kolmogorov-Smirnov test for equal distributions). As the growth in school’s grades does not show differences by gender, we conclude that temporal trends do not influence the gender gaps we obtained comparing the 10^th^ grade SIMCE test with the PSU test.

Additionally, in Appendix S4, we show the estimation results of the three models for Mathematics and Language, with the 10^th^ and 12^th^ year school’s grades as the dependent variable replacing 10^th^ grade SIMCE and the PSU test scores. If the gender gaps observed in the 10^th^ grade SIMCE and the PSU tests were explained by temporal trends rather than differences between both tests, we should be able to find the same gaps in school’s grades. Our analysis shows that in Mathematics, there is no gender gap in 10^th^ and 12^th^ school’s grades. In Language, there are gender gaps in favor of women in both grade levels. Consequently, the differences in the gaps observed in SIMCE (noncompetitive test) and PSU (competitive test) cannot be explained by temporal trends in students’ academic performance.

## Discussion

This research studies the extent to which high-stakes competitive tests affect gender performance gaps in Mathematics and Language tests. To do this, we estimated models to predict student results in two national standardized tests of Mathematics and Language in Chile. The first test does not affect the educational trajectory of the students -and it is the one in which women surpass men in Language, and men surpass women in Mathematics-, the second test determines access to the most selective universities in Chile. We utilized data from six cohorts of different gender twins who took these tests. This empirical strategy makes it possible to control for the observed and unobserved household characteristics -through household fixed effects- while analyzing the effect of competitive tests on subjects where the gender gap has opposite signs. Our results show that the gender gap in performance differs between the 10th grade non-competitive SIMCE test and the competitive PSU test, which students take two years after the SIMCE test. In particular, in Mathematics, the gender gap in favor of men in the 10^th^ grade SIMCE test increases in the PSU test. In the case of Language, the gender gap in favor of women that is observed in the noncompetitive test is reversed in the competitive test. We did not find differences in the gender gap when analyzing school grades between grades 10^th^ and 12^th^. Therefore, we did not find evidence of a temporal trend that could explain the improvements in the performance of men in the tests. In summary, our findings show that competitive tests affect the performance of women, who obtain lower results than men (between 0.12 and 0.14 standard deviations less) and lower scores than those they obtained in noncompetitive tests. Following the literature, this gender gap could be related to women´s greater risk aversion, lower self-confidence, and less preference for competition, as well as the effect of answering a test under time pressure.

In addition, the analysis of the models that incorporate interactions between the variable Women and previous performance shows -in the case of Mathematics- that the greatest gender gap (in the competitive PSU test) occurs mainly among students with medium-high to high academic achievement. In these groups, men tend to outperform women in the PSU test, with the magnitude of the gender gap being greater in the PSU than in the SIMCE test. Gender stereotyping could explain this difference. First, negatively stereotyped populations, like women in Mathematics, often obtain results that underestimate their cognitive abilities, due to a working memory overload caused by stress [23,72]. The importance of the PSU Mathematics test for university selection is probably greater for high-achieving students, which may lead to higher test anxiety and in turn, explain why high-achieving women are more affected. Second, stereotypes could affect the self-confidence of high-achieving women. According to Niederle & Vesterlund [14], gender differences in self-confidence and a lower preference for competition play an important role in Mathematics, and even more so in the right tail of the achievement distribution. The authors argue that women have little faith in their own mathematical abilities due to extensive gender stereotyping by parents and teachers. Preckel et al. [63] found that this lower self-perception is exacerbated among the most able women, and could be the result of a greater awareness of social expectations linked to their aptitudes. Given the relevance of the Mathematics PSU test for university selection, the lack of self-confidence could negatively affect their results.

In the case of Language, we found no evidence that the gender gap varies as a function of the level of academic performance. In contrast, the negative effect of the Language competitive test on women seems to be present throughout the achievement distribution. In addition, the fact that competitive tests affect the performance of women in Language indicates that the effect of the competitive test goes beyond Mathematics. It also shows that men, who might be negatively stereotyped in Language, may not be affected by stereotype threat in this context.

It is worth noting that the estimates made for the total population, without household fixed effects, show the same patterns observed for the twin population, although with some differences in magnitudes. So, the negative effect of competitive tests on women is not exclusive to twin population but rather a phenomenon that can be generalized to the entire student population.

These research results are relevant to public policy. When translated into the PSU 150 to 850 scale, 0.12 and 0.14 standard deviations less correspond to 13.2 and 15.4 fewer PSU points, respectively. If women with similar abilities to men underperform men by that magnitude, the result will hurt their chances of getting into the desired college major. An example shows the importance of these score differences in Chile. In 2020, a student with 800 points in all the PSU tests, and equivalent school grades, would have been in 82^nd^ place among the applicants for the most selective program in the country (e.g., Medicine at a prestigious university), which had 92 places available. On the other hand, someone with 15.4 points less in all the PSU tests, but with the same school grades, would be ranked 155^th^ in the same program, and would not be selected. Not only do university entrance opportunities depend on the results of the competitive PSU test, but also access to scholarships and financial assistance. So, any implicit bias in this test against women affects their educational opportunities.

It could be argued that performing well in competitive environments is a professional requirement. However, university entrance exams are not explicitly designed to measure this skill, but rather the examinees’ knowledge and skills established by the national curriculum. Even if the ability to tackle competitive environments is a desirable professional competence (and perhaps should be part of the student selection process) there are other equally or more important skills to be taken into account -such as teamwork and oral communication skills- where there may not be a gender gap favoring men. In addition, as this specifically affects women with high performance in Mathematics, it could limit their access to STEM careers, which is a loss of talent for these disciplines.

These results are relevant and have important consequences. In Chile, women represent 55 percent of current university enrollment [95]. They are, however, underrepresented in STEM fields and overrepresented in humanities, health, and education [3]. The overrepresentation of one gender compared to the other in the fields of study could explain, in part, the gender segregation in the labor market [4] and, consequently, the fact that women access jobs with lower expectations of employability and wages. Thus, gender segregation in these fields contributes to the gender wage gap, as holders of STEM degrees tend to have higher salaries [96]. The positive causal impact of advanced Mathematics on earnings is pronounced [97].

Our argument is that the gender inequalities found in the educational system and, subsequently, in the labor market are partly due to gender stereotypes generated during upbringing and school education. These stereotypes reduce the employment opportunities of women. The finding has a social impact, since if women with high skills in Mathematics are implicitly or explicitly excluded from fields such as STEM, societies are wasting valuable human capital for some of the most productive jobs [98], not to mention the positive benefits of gender diversity. Specifically, research shows that diverse environments encourage new questions, new approaches, and better answers [99].

In terms of public policy, the gender gap favoring men in competitive tests creates short- and long-term challenges. In the short term, it is necessary to think on how to design university access mechanisms that take into account the negative effect of competitive tests on high-performing women which, as research shows, can have better results than men in their undergraduate STEM courses [100]. So, test designs to reduce test anxiety and the risk (associated with the test) is likely to help limit these gaps. In fact, Chile has begun to experiment with a number of new approaches to this problem. Before 2015, the PSU test included a penalty for answering a question incorrectly; after the elimination of this penalty, the gender gaps in the PSU were reduced [101]. Recently, the Committee of Rectors of Universities that oversees university entrance criteria decided, starting in 2022, to offer two opportunities a year to take the university entrance exam, in the middle and end of the calendar year.

It is also important to analyze university access policies with gender criteria, for example, gender quotas for certain university degrees. This type of policy has two advantages. First, it helps offset the negative effect of competitive testing on female candidates. Second, in masculinized (feminized) fields, it sends the message that women (men) are welcome in these careers. It is important to note that gender quotas do not imply accepting unprepared students because universities can still require the necessary academic standards to ensure that their students have the required level of knowledge and/or skills. In fact, in the case of Chile, selective institutions are in high demand; thus, current cutoff scores usually depend on the number of vacancies offered, since a large number of applicants meet the minimum requirements.

This policy has already been implemented in the engineering faculty of one of the most prestigious universities in Chile, initially 40 additional vacancies were created and, today, 70 exclusive vacancies for women. These spots are allocated based on applicants’ PSU test scores and school grades, and are awarded to women who applied to study engineering as their first choice but fell just below the cutoff score [102]. As a result of this policy, the total percentage of women studying engineering at that university increased from 18% in 2014 to 28% in 2021, and the number of high-achieving women applying to engineering with scores above the cutoff also increased. More importantly, the gender quota has increased the participation of women in tasks where high-performing women were less willing to compete [102]. Gender quotas can break the inertia of institutions by changing the dynamics in areas previously considered reserved for men. Therefore, they can progressively contribute to weakening gender stereotypes, increase women’s confidence in their abilities, and lessen the impact of stereotype threat in competitive events.

In the longer term, it is necessary to avoid gender stereotypes in parenting and school education. To this end, it is also important to include the gender issue in pre-service and in-service teacher preparation curricula, as well as to avoid stereotypes in textbooks and school curricula. In addition, exposing boys and girls to alternative role models who do not respond to gender stereotypes allows them to open their minds and change stereotyped beliefs [103]. For example, gender stereotypes can be weakened by introducing women scientists and men who work in health or education to students in schools. The stereotypes are also undermined by developing policies that promote gender-neutral advertising for college majors.

Finally, it is worth noting that most of our analysis are based on the results of national standardized tests, since the scores assigned to students on these tests are not affected by gender bias. Nonetheless, in assessing possible temporal trends in student performance, which could explain observed differences between SIMCE and PSU test scores, we used school grade data. Since grades are given by teachers and are not anonymous, they can be biased; for example, by teacher stereotypes. We acknowledge that this is a limitation of our study because there may be time-varying gender biases that affect school grades. In such a case, our analysis may not be fully representative of temporal trends in student performance.

## Supporting information

S1 Appendix Estimates using the total population. (PDF)

S2 Appendix Classification of students according to their previous academic achievement. (PDF)

S3 Appendix Estimates using alternative classifications of students. (PDF)

S4 Appendix Additional estimates to study temporal trends that could affect estimations. (PDF)
