# Supplementary material for: Gender gaps in Mathematics and Language: The bias of competitive achievement tests
Source: PLoS One. 2023 Mar 22;18(3):e0283384. doi: 10.1371/journal.pone.0283384 (PMC10032501; doi:10.1371/journal.pone.0283384)
Supplement: S1 Appendix — (PDF) [file pone.0283384.s001.pdf]

S1 Appendix: Estimates using the total population

Although the use of data from pairs of different gender twins has the advantage of controlling for observed and unobserved household characteristics, twin populations may have some specific characteristics that differ from the total population. To test whether the results obtained from working with twins hold for the total population, we make two additional estimates. First, we estimate the three variants of the model presented in the main text without including household fixed effect. Secondly, we estimate the same variants of the model, but including two variables to control for household characteristics, that is, household income (in thousands of CLP 2020) and the maximum educational level of the parents (years of schooling).

Gender gap in Mathematics

Table S1 shows the estimation of the three variants of the model without household fixed effects, to predict both 10th grade Mathematics SIMCE test results and PSU test results for the total population. Table S2 shows the same estimations but including household control variables. They show similar patterns to those observed in the twin population, but with some small differences in magnitude and with greater statistical significance (see Table 4 in the main text):

- Estimates from model (1) show that women have lower performance on both tests but the gender gap is larger on the competitive test. Note that the difference in the size of the effect for the female variable between the SIMCE and PSU tests is slightly smaller in the total population than in the twin population estimates.
- In Model (2), the estimates of the interaction between the gender variable and the previous score in the SIMCE test show that women and men with higher previous scores obtain better SIMCE scores in 10th grade and in the PSU. In addition, in both tests there are statistically significant differences between men and women (P-value <0.00). Although in both cases the gender gap in the total population is smaller than in the twin population, the pattern is the same, the gender gap is greater in the PSU test than in the SIMCE test.
- Model (3) includes the interaction between gender and the student's performance group, which we calculate using the scores of the 4th grade SIMCE test. Table S3 presents the estimated coefficients in model 3 of men and women who belong to the same achievement group and compares the gap between them. We found that there are statistically significant gender differences between tests and for each performance group. However, only for the high or medium-high performance groups, the gender gap in favor of men grows in the PSU test.

In general, the estimates for the total population show results consistent with the findings for the twin population. Women are negatively affected by competitive testing; also, the high and medium-high performance groups drive the effect.

Table S1. Estimated coefficient. Models to predict Mathematics performance in competitive (PSU) and noncompetitive (10th grade SIMCE) tests.
Total Population without household control variables

	10th grade SIMCE			PSU		
	**Model 1**	**Model 2**	**Model 3**	**Model 1**	**Model 2**	**Model 3**
**Women**	-0.094***	-0.101***		-0.148***	-0.130***	
**Men * Previous mathematics SIMCE score[1]**		0.275***			0.421***	
**Women * Previous mathematics SIMCE score[1]**		0.292***			0.371***	
**Men * Medium-low achievement**			0.235***			0.071***
**Men * Medium-high achievement**			0.472***			0.279***
**Men * High achievement**			0.704***			0.639***
**Women * Low achievement**			-0.167***			-0.115***
**Women * Medium- low achievement**			0.079***			-0.080***
**Women * Medium- high achievement**			0.351***			0.099***
**Women * High achievement**			0.591***			0.429***
**Previous mathematics SIMCE score[1]**	0.284***			0.393***		
**Previous language SIMCE score[1]**	0.027***	0.027***		0.068***	0.068***	
**10th grade mathematics SIMCE school score [1]**	0.486***	0.486***	0.516***	0.333***	0.333***	0.520***
**Mathematics grades [2]**	0.323***	0.323***	0.352***	0.242***	0.241***	0.346***
**Attendance rate (%)**	0.005***	0.005***	0.004***	0.002***	0.002***	0.004***
**Students with university entrance expectation [3]**	0.126***	0.126***	0.131***	0.160***	0.158***	0.197***
**Constant**	-0.525***	-0.519***	-0.833***	-0.401***	-0.417***	-0.821***
**N**	595,030	595,030	595,030	595,030	595,030	595,030
**Adjusted R^2**	0.664	0.664	0.650	0.625	0.626	0.597

[1] SIMCE and PSU variables were standardized to a distribution with mean equal to zero, and standard deviation equal to 1—standardization made by cohort.
[2] School grades were standardized to a distribution with mean equal to zero, and standard deviation equal to 1—standardization made by cohort and school.
[3] Students with university entrance expectation is a dummy variable. Its value equals 1 when the student in 10th grade expected to attend the university, and 0 otherwise.
* $p<0.05$, ** $p<0.01$, *** $p<0.001$.

Table S2. Estimated coefficient. Models to predict Mathematics performance in competitive (PSU) and noncompetitive (10th grade SIMCE) tests. Total Population with household control variables

	10th grade SIMCE			PSU		
	**Model 1**	**Model 2**	**Model 3**	**Model 1**	**Model 2**	**Model 3**
**Women**	-0.093***	-0.100***		-0.132***	-0.112***	
**Men * Previous mathematics SIMCE score[1]**		0.274***			0.421***	
**Women * Previous mathematics SIMCE score[1]**		0.290***			0.369***	
**Men * Medium-low achievement**			0.235***			0.008***
**Men * Medium-high achievement**			0.474***			0.285***
**Men * High achievement**			0.702***			0.625***
**Women * Low achievement**			-0.164***			-0.099***
**Women * Medium- low achievement**			0.084***			-0.056***
**Women * Medium- high achievement**			0.353***			0.120***
**Women * High achievement**			0.588***			0.428***
**Previous mathematics SIMCE score[1]**	0.283***			0.392***		
**Previous language SIMCE score[1]**	0.026***	0.026***		0.074***	0.074***	
**10th grade mathematics SIMCE school score [1]**	0.471***	0.471***	0.496***	0.239***	0.239***	0.437***
**Mathematics grades [2]**	0.324***	0.323***	0.353***	0.241***	0.239***	0.348***
**Attendance rate (%)**	0.005***	0.004***	0.004***	0.003***	0.003***	0.004***
**Students with university entrance expectation[3]**	0.119***	0.119***	0.122***	0.127***	0.125***	0.169***
**Household-income (thousands of CLP 2020)**	0.000***	0.000***	0.000***	0.000***	0.000***	0.000***
**Parents' educational level (mean schooling years)**	0.007***	0.007***	0.008***	0.016***	0.016***	0.016***
**Constant**	-0.581***	-0.575***	-0.910***	-0.716***	-0.736***	-1.115***
**N**	507,257	507,257	507,257	507,257	507,257	507,257
**Adjusted R^2**	0.668	0.668	0.653	0.638	0.639	0.607

[1] SIMCE and PSU variables were standardized to a distribution with mean equal to zero, and standard deviation equal to 1—standardization made by cohort.
[2] School grades were standardized to a distribution with mean equal to zero, and standard deviation equal to 1—standardization made by cohort and school.
[3] Students with university entrance expectation is a dummy variable. Its value equals 1 when the student in 10th grade expected to attend the university, and 0 otherwise.

* $p<0.05$, ** $p<0.01$, *** $p<0.001$.

Table S3. Gender gaps comparison for different levels of previous achievement. Estimations based on Model (3) for Mathematics area for the total population

A. Total Population without household control variables

		Coefficient			
	**Achievement group**	**Men**	**Women**	**Gap**	**p-value***
**SIMCE**	**Low**	0.00	-0.167	0.167	0.00
	**Medium-Low**	0.235	0.079	0.156	0.00
	**Medium-High**	0.472	0.351	0.121	0.00
	**High**	0.704	0.591	0.113	0.00
**PSU**	**Low**	0.00	-0.115	0.115	0.00
	**Medium-Low**	0.071	-0.08	0.151	0.00
	**Medium-High**	0.279	0.099	0.18	0.00
	**High**	0.639	0.429	0.21	0.00

B. Total Population with household control variables

		Coefficient			
	**Achievement group**	**Men**	**Women**	**Gap**	**p-value***
**SIMCE**	**Low**	0.000	-0.164	0.164	0.00
	**Medium-Low**	0.235	0.084	0.151	0.00
	**Medium-High**	0.474	0.353	0.121	0.00
	**High**	0.702	0.588	0.114	0.00
**PSU**	**Low**	0.000	-0.099	0.099	0.00
	**Medium-Low**	0.008	-0.056	0.064	0.00
	**Medium-High**	0.285	0.120	0.165	0.00
	**High**	0.625	0.428	0.197	0.00

*p-value in test F in order to prove the null hypothesis of coefficient equality.

Gender gap in language

Table S4 and Table S5 show the estimation of the three variants of the model used to predict both the 10th grade Language SIMCE and the PSU test for the total population with and without controlling for household characteristics. These results are similar to those obtained with the twin population. However, there are some differences in the magnitude of the estimated coefficients and, due to the large number of observations when considering the total population, statistically more significant effects (see Table 6 in the main text):

- The estimates from model (1) show that women outperform men in the SIMCE test, but the opposite occurs in the PSU test. However, the difference in the magnitude of the effect of the Women variable between the SIMCE and PSU tests is slightly smaller in the total population than in the estimates considering only the twin population.
- In Model (2), the estimated coefficients of the interaction between the gender variable and the previous score in the 4th grade SIMCE test show that women and men with higher previous scores obtain better scores in both the 10th grade SIMCE test and the PSU test. In both cases, the gender gap for the total population is smaller than in the twin population, although it is statistically significant (P-value <0.00).

- Model (3) includes the interaction between gender and the performance group of the student, which we calculate using the scores of the 4th grade SIMCE test. Table S6 presents the estimated coefficients of men and women who belong to the same achievement group and compares the gap between them. In both tests and for each performance group, there are statistically significant gender differences, but the gender gap differs between the tests. While in the SIMCE test men of a certain performance group have lower result than women of the same group, the opposite happens in the PSU test. In addition, within each performance group, the changes in the magnitudes of the gender gap between the SIMCE and the PSU test are quite similar.

In summary, the results for the total population are consistent with the findings for the twin population: women are negatively affected by competitive testing, and this effect is not driven by a specific level of performance.

Table S4. Estimated coefficient. Models to predict Language performance in competitive (PSU) and noncompetitive (10th grade SIMCE) tests. Total Population without household control variables

	10th grade SIMCE			PSU		
	**Model 1**	**Model 2**	**Model 3**	**Model 1**	**Model 2**	**Model 3**
**Women**	0.114***	0.111***		-0.110***	-0.122***	
**Men * Previous language SIMCE score**[1]		0.299***			0.309***	
**Women * Previous language SIMCE score**[1]		0.308***			0.350***	
**Men * Medium-low achievement**			0.288***			0.323***
**Men * Medium- high achievement**			0.636***			0.731***
**Men * High achievement**			1.020***			1.222***
**Women * Low achievement**			0.092***			-0.116***
**Women * Medium-low achievement**			0.417***			0.211***
**Women * Medium-high achievement**			0.780***			0.625***
**Woman * High achievement**			1.142***			1.108***
**Previous mathematics SIMCE score**[1]	0.130***	0.130***		0.307***	0.306***	
**Previous language SIMCE score**[1]	0.304***			0.329***		
**10th grade Language SIMCE school score**[1]	0.290***	0.290***	0.308***	0.179***	0.178***	0.360***
**Language grades** [2]	0.241***	0.241***	0.262***	0.190***	0.190***	0.270***
**Attendance rate (%)**	0.002***	0.002***	0.002***	-0.002***	-0.002***	0.001***
**Students with university entrance expectation** [3]	0.123***	0.123***	0.129***	0.248***	0.249***	0.289***
**Constant**	-0.362***	-0.359***	-0.845***	-0.105***	-0.098***	-1.044***
**N**	578,900	578,900	578,900	578,900	578,900	578,900
**Adjusted R^2**	0.45	0.45	0.432	0.61	0.61	0.587

[1] SIMCE and PSU variables were standardized to a distribution with mean equal to zero, and standard deviation equal to 1—standardization made by cohort.
[2] School grades were standardized to a distribution with mean equal to zero, and standard deviation equal to 1—standardization made by cohort and school.
[3] Students with university entrance expectation is a dummy variable. Its value equals 1 when the student in 10th grade expected to attend the university, and 0 otherwise.

* p<0.05, ** p<0.01, *** p<0.001

Table S5. Estimated coefficient. Models to predict Language performance in competitive (PSU) and noncompetitive (10th grade SIMCE) tests. Total Population with household control variables

	10th grade SIMCE			PSU		
	**Model 1**	**Model 2**	**Model 3**	**Model 1**	**Model 2**	**Model 3**
**Women**	0.111***	0.108***		-0.091***	-0.102***	
**Men * Previous language SIMCE score[1]**		0.300***			0.330***	
**Women * Previous language SIMCE score[1]**		0.308***			0.364***	
**Men * Medium-low achievement**			0.292***			0.320***
**Men * Medium- high achievement**			0.641***			0.715***
**Men * High achievement**			1.026***			1.165***
**Women * Low achievement**			0.094***			-0.086***
**Women * Medium-low achievement**			0.420***			0.236***
**Women * Medium-high achievement**			0.784***			0.631***
**Women * High achievement**			1.142***			1.072***
**Previous mathematics SIMCE score[1]**	0.130***	0.130***		0.268***	0.268***	
**Previous language SIMCE score[1]**	0.304***			0.347***		
**10th grade Language SIMCE school score[1]**	0.283***	0.283***	0.298***	0.107***	0.107***	0.287***
**Language grades [2]**	0.239***	0.239***	0.261***	0.195***	0.195***	0.278***
**Attendance rate (%)**	0.001***	0.001***	0.001***	-0.001***	-0.001***	0.001***
**Students with university entrance expectation[3]**	0.117***	0.118***	0.122***	0.193***	0.194***	0.234***
**Household-income (thousands of CLP 2020)**	-0.000	-0.000	0.000	0.000***	0.000***	0.000***
**Parents' educational level (mean schooling years)**	0.006***	0.006***	0.007***	0.032***	0.032***	0.031***
**Constant**	-0.361***	-0.359***	-0.863***	-0.628***	-0.620***	-1.483***
**N**	493961	493962	493963	493964	493965	493966
**Adjusted R^2**	0.456	0.456	0.438	0.637	0.637	0.608

[1] SIMCE and PSU variables were standardized to a distribution with mean equal to zero, and standard deviation equal to 1—standardization made by cohort.
[2] School grades were standardized to a distribution with mean equal to zero, and standard deviation equal to 1—standardization made by cohort and school.
[3] Students with university entrance expectation is a dummy variable. Its value equals 1 when the student in 10th grade expected to attend the university and 0 otherwise.
* $p<0.05$, ** $p<0.01$, *** $p<0.001$

Table S6. Gender gaps comparison for different levels of previous achievement. Estimations based on Model (3) for Language area for the total population

A. Total population without household control variables

	Coefficient				
	**Achievement group**	**Men**	**Women**	**Gap**	**p-value***
**SIMCE**	**Low**	0	0.092	-0.092	0.00
	**Medium-Low**	0.288		-0.129	0.00
	**Medium-High**	0.636	0.780	-0.144	0.00
	**High**	1.020	1.142	-0.122	0.00
**PSU**	**Low**	0	-0.116	0.116	0.00
	**Medium-Low**	0.323	0.211	0.112	0.00
	**Medium-High**	0.731	0.625	0.106	0.00
	**High**	1.222	1.108	0.114	0.00

C. Total population with household control variables

	Coefficient				
	**Achievement group**	**Men**	**Women**	**Gap**	**p-value***
**SIMCE**	**Low**	0.000	0.094	-0.094	0.00
	**Medium-Low**	0.292	0.420	-0.128	0.00
	**Medium-High**	0.641	0.784	-0.143	0.00
	**High**	1.026	1.142	-0.116	0.00
**PSU**	**Low**	0.000	-0.086	0.086	0.00
	**Medium-Low**	0.320	0.236	0.084	0.00
	**Medium-High**	0.715	0.631	0.084	0.00
	**High**	1.165	1.072	0.093	0.00

*Value-P in F test in order to prove the null hypothesis of coefficient equality.
