# Supplementary material for: Gender gaps in Mathematics and Language: The bias of competitive achievement tests
Source: PLoS One. 2023 Mar 22;18(3):e0283384. doi: 10.1371/journal.pone.0283384 (PMC10032501; doi:10.1371/journal.pone.0283384)
Supplement: S2 Appendix — (PDF) [file pone.0283384.s002.pdf]

S2 Appendix: Classification of students according to their previous academic achievement

Given that students in different achievement groups may react differently to competitive tests, we grouped them according to their performance on the 4th grade SIMCE math and language test. First, we use the Euclidean distance k-means clustering methodology to identify 2, 3, 4, and 5 clusters. Fig 1, which is featured in the main text, plots students as they are classified into four groups, based on their academic performance in math and language. Fig A1 of this annex shows the same information when students are classified into 2, 3, and 5 groups.

Figure A1. Students' achievement groups. Alternative groups of students generated using k-means algorithm

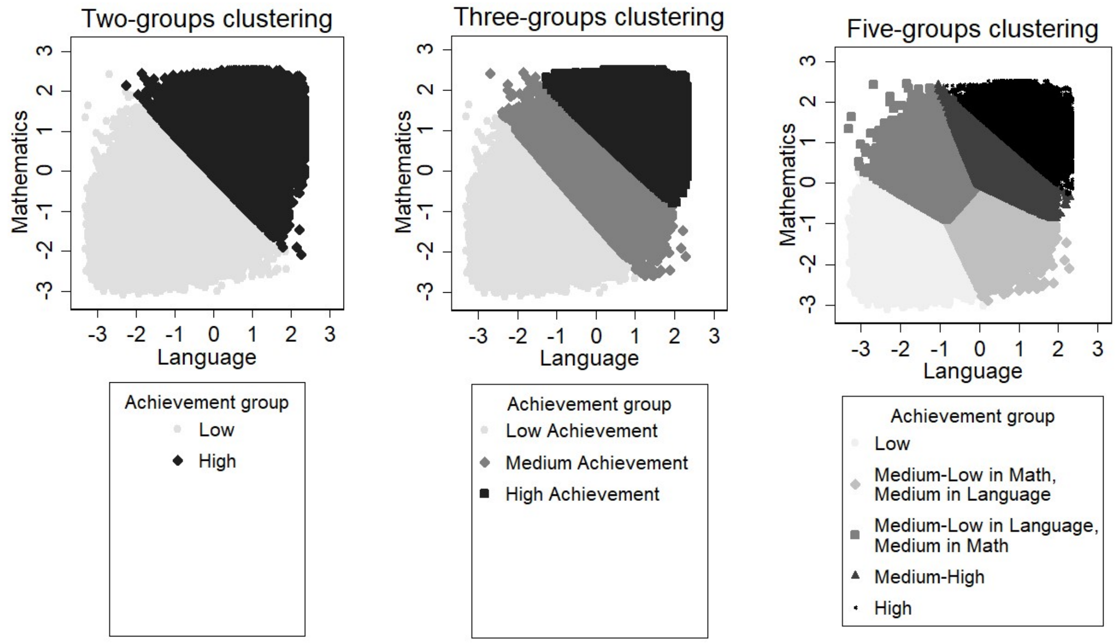

Note: x- axis and y-axis show 4th grade SIMCE test scores on Language and Mathematics, respectively.

Two analyzes are performed to select the most suitable number of groups. First, elbow analysis [1]. Fig A2 shows the within-cluster sum of squares, which measures the within-cluster variance using a different number of groups. With this methodology, it is not possible to identify a clear change in the trend, since the number of optimal groups is not evident. However, it is possible to identify that a proper number of groups must be in the range between 3 and 5.

Second, to compare how appropriate the different groupings are, we perform the silhouette analysis [2], which estimates how close the students are to each cluster with respect to the other possible clusters. For each student, a silhouette coefficient is estimated -with a range [-1,1], where high values indicate that the student is more similar to their group than to the other groups. Fig A3 shows this coefficient for all students – according to their cluster membership – considering 2, 3, 4, and 5 clusters. It shows that, for all possible groupings (2, 3, 4 and 5 clusters), the students are more related to their group than to others. Taking into account these last results, in the main text we show the grouping using four groups of students according to their performance, since it allows a balance between a simple classification and a good representation of the diversity of student performance. Regardless, Appendix S3 shows the results using the other clustering.

Figure A2.Choosing the number of clusters: Elbow analysis

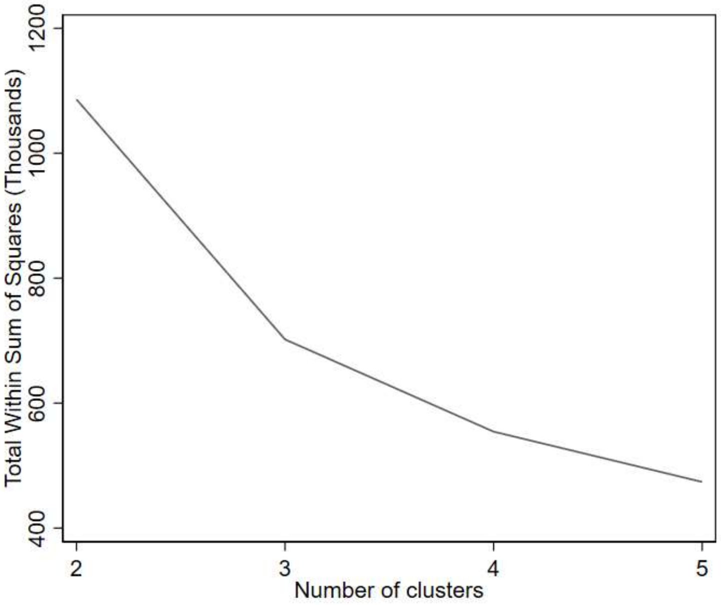

Figure A3. Choosing the number of groups: Silhouette analysis

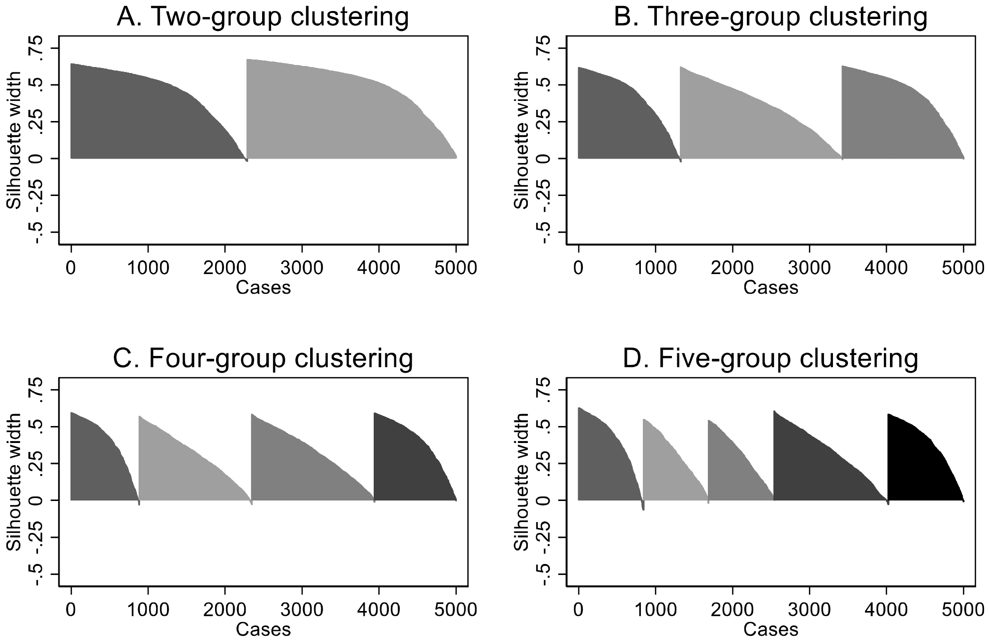

Note: Plot made with a random sample of 5000 students

Besides the classifications using the k-means clustering algorithm, we consider two other possible grouping mechanisms using the 4th grade SIMCE test scores. The first classifies students into 3

groups, according to the average between their Language and Mathematics 4th grade SIMCE scores (called the Three-groups classification):[1]

1. Low performance: Average SIMCE scores below -0.5.
2. Medium performance: Average SIMCE scores between -0.5 and 0.5
3. High performance: Average SIMCE scores above 0.5.

The second classification consists of four different groups of students, according to their performance in the subjects (called a four-group classification). Note that since we are using standardized test scores, the average SIMCE test score is 0. Therefore, we use 0 as the cutoff point to divide the population into the following groups:

1. Low performance: 4th grade SIMCE Scores in Mathematics and Language below or equal to 0.
2. High Performance in Language, Low performance in Mathematics: 4th grade SIMCE scores in Mathematics below or equal to 0 and 4th grade SIMCE scores in Language above 0.
3. High performance in Mathematics, low performance in Language: 4th grade SIMCE test scores in Language below or equal to 0 and 4th grade SIMCE test scores in Mathematics above 0.
4. High performance: 4th grade SIMCE test scores in Mathematics and Language above 0.

Fig A4 represents both forms of grouping. S3 Appendix shows the estimates using these alternative classifications.

Figure A4. Alternative groups of students using different classifications

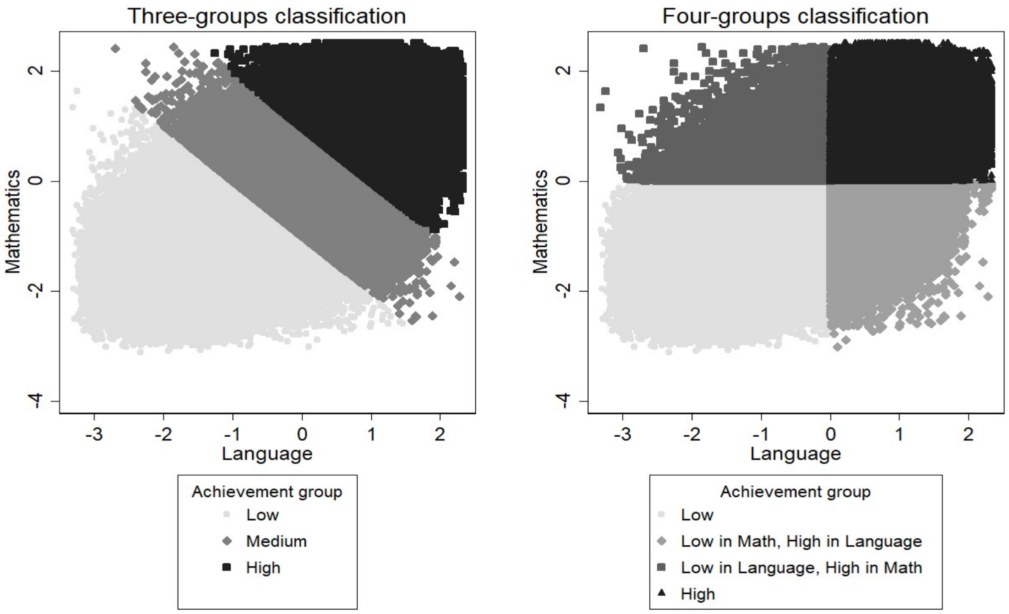

Note: x- axis and y-axis show 4th grade SIMCE test scores on Language and Mathematics, respectively.

---

[1] The range -0.5 and 0.5 was used as a cutoff because it allows the division of the student population into three groups of similar size. The proportion of students in groups 1, 2, and 3 are 31%, 37%, and 32%, respectively.

References

1. Thorndike RL. Who belongs in the family? Psychometrika. 1953;14(4).
2. Rousseeuw PJ. Silhouettes: a graphical aid to the interpretation and validation of cluster analysis. Journal of Computational and Applied Mathematics. 1987;20:53-65.
