# Supplementary material for: Gender gaps in Mathematics and Language: The bias of competitive achievement tests
Source: PLoS One. 2023 Mar 22;18(3):e0283384. doi: 10.1371/journal.pone.0283384 (PMC10032501; doi:10.1371/journal.pone.0283384)
Supplement: S3 Appendix — (PDF) [file pone.0283384.s003.pdf]

S3 Appendix: Estimates using alternative classifications of students

Gender gap in Mathematics

To ensure that the third variation of the model provides robust estimates and is not affected by the classification of students in achievement groups, we re-estimate the model using the alternative classifications of students. Table S7 shows the estimated coefficients for men and women of each achievement group, using the rankings generated with the k-means clustering methodology. Table S8 shows the same results, but for the two additional clusters described in Appendix S2.

The results show, as the previous estimations, that high-achieving women are the most negatively affected by the competitive tests. Specifically:

1. When using the two-group clustering, in the high achievement group, men have higher 10th grade SIMCE and PSU scores compared to women, but the gap is higher in the PSU test.
2. When using the three-group clustering, in the medium and high achievement groups, men have higher 10th grade SIMCE and PSU scores compared to women, but the gap is higher in the PSU test.
3. When using the five-group clustering, in the medium and high achievement groups, men have higher 10th grade SIMCE and PSU scores compared to women, but the gap is higher in the PSU test. Furthermore, in the 10th grade SIMCE test, there are no significant differences between men and women from medium-low achievement group in Language and medium achievement group in Mathematics. However, there are statistically significant differences between men and women from this group in the PSU scores.
4. When using the additional three-group classification, in the medium and high achievement groups, men have higher 10th grade SIMCE and PSU scores compared to women, but the gap is higher in the PSU test.
5. When using the additional four-group classification, in the high achievement group, men have higher 10th grade SIMCE and PSU scores compared to women, but the gap is higher in the PSU test. In addition, in the 10th grade SIMCE test, there are no significant differences between men and women in the other groups. However, in the PSU, there are significant differences between men and women from the group with medium-low achievement in Language and medium achievement in Mathematics.

Table S7. Gender gaps comparison for different levels of previous performance. Estimations based on Model (3) for Mathematics, using alternative student classifications (1)

**A. Two groups-clustering (k-means)**					
		**Coefficient**			
	**Achievement group**	**Men**	**Women**	**Gap**	**p-value***
**SIMCE**	**Low**	0.00	-0.20	0.20	0.77
	**High**	0.28	0.16	0.12	0.00
**PSU**	**Low**	0.00	-0.11	0.11	0.25
	**High**	0.31	0.11	0.20	0.00
**B. Three- groups-clustering (k-means)**					
		**Coefficient**			
	**Achievement group**	**Men**	**Women**	**Gap**	**p-value***
**SIMCE**	**Low**	0.00	0.00	0.00	0.97
	**Medium**	0.29	0.20	0.09	0.05
	**High**	0.41	0.30	0.11	0.00
**PSU**	**Low**	0.00	0.00	0.00	1.00
	**Medium**	0.31	0.09	0.22	0.00
	**High**	0.46	0.29	0.17	0.00
**C. Five groups-clustering (k-means)**					
		**Coefficient**			
	**Achievement group**	**Men**	**Women**	**Gap**	**p-value***
**SIMCE**	**Low**	0.00	0.09	-0.09	0.60
	**Medium-Low in Math, Medium in Language**	0.31	0.27	0.04	0.66
	**Medium-Low in Language, Medium in Math**	0.35	0.23	0.12	0.21
	**Medium-High**	0.49	0.38	0.11	0.03
	**High**	0.66	0.55	0.11	0.03
**PSU**	**Low**	0.00	0.03	-0.03	0.89
	**Medium-Low in Math, Medium in Language**	0.11	0.01	0.10	0.56
	**Medium-Low in Language, Medium in Math**	0.28	0.00	0.28	0.02
	**Medium-High**	0.35	0.19	0.16	0.00
	**High**	0.54	0.34	0.20	0.00

Estimates made by controlling for the school's mean 10th grade Mathematics SIMCE test score, attendance rate, Mathematics grades, expectation of students to enter the university, and household fixed effects.

* p-value in the F test to prove the null hypothesis of equality of coefficients.

Table S8. Gender gaps comparison for different levels of previous performance. Estimations based on Model (3) for Mathematics, using alternative student classifications (2)					
**A. Three groups-classification**					
		**Coefficient**			
	**Achievement group**	**Men**	**Women**	**Gap**	**p-value***
**SIMCE**	**Low**	0.00	-0.01	0.01	0.95
	**Medium**	0.25	0.14	0.11	0.04
	**High**	0.37	0.26	0.11	0.00
**PSU**	**Low**	0.00	0.01	-0.01	0.96
	**Medium**	0.36	0.13	0.23	0.00
	**High**	0.49	0.31	0.18	0.00
**B. Four groups-classification**					
		**Coefficient**			
	**Achievement group**	**Men**	**Women**	**Gap**	**p-value***
**SIMCE**	**Low**	0.00	0.04	-0.04	0.67
	**Low in Math, High in Language**	0.26	0.14	0.12	0.27
	**Low in Language High in Math**	0.27	0.14	0.13	0.25
	**High**	0.38	0.26	0.12	0.00
**PSU**	**Low**	0.00	-0.12	0.12	0.59
	**Low in Math, High in Language**	0.15	0.04	0.11	0.35
	**Low in Language High in Math**	0.40	0.20	0.20	0.00
	**High**	0.55	0.37	0.18	0.00

Estimates made by controlling for the school's mean 10th grade Mathematics SIMCE test score, attendance rate, Mathematics grades, expectation of students to enter the university, and household fixed effects.
* p-value in the F test to prove the null hypothesis of equality of coefficients.

Gender gap in Language

To ensure that the third variation of the model provides robust estimations, and is not affected by the classification of students into achievement groups, we re-estimate the model using the alternative classifications of students. Table S9 shows the estimated coefficients for men and women of each achievement group, using the classifications generated with the k-means clustering methodology. Table S10 shows the same results, but for the two additional clusters described in Appendix S2.

We do not find consistent results that show that gender gaps are generated only at a certain level of performance. Therefore, it is not possible to affirm that there is an interaction between gender and performance that explains the lower scores of women -compared to men- in the competitive Language tests. Specifically, we found that:

1. Using the two-group clustering, there are no statistically significant differences in performance between men and women in the low-performing group. However, there is a positive gender effect for high-performing men in the PSU; with 95% confidence, men have higher scores than women in the PSU test, this does not happen in the 10th grade SIMCE test.

2. Using the three-group clustering, we do not find statistically significant differences in PSU or 10th grade SIMCE score between men and women from any performing group.
3. Using the five-group clustering, there are no statistically significant differences (at 95% confidence level) in performance between men and women from any achievement group.
4. Using the additional three-group classification, there are no statistically significant differences in performance between men and women from high or low achievement groups. However, there is a positive effect for men from the medium achievement group in the competitive tests: With a 95% confidence level, they have higher PSU score than women, but there are no differences between them in the 10th grade SIMCE score.
5. Using the additional four-group classification, there are no statistically significant differences in performance between men and women except in one group. With a 95% of confidence level, there is a positive effect for men from the high achievement group, as they obtain higher PSU scores than women, while there are no differences in the 10th grade SIMCE score.

Table S9. Gender gaps comparison for different levels of previous performance. Estimations based on Model (3) for Language, using alternative student classifications (1)

A. Two groups-clustering (k-means)

		Coefficient			
	**Achievement group**	**Men**	**Women**	**Gap**	**p-value***
**SIMCE**	**Low**	0.00	0.16	-0.16	0.05
	**High**	0.55	0.65	-0.10	0.05
**PSU**	**Low**	0.00	-0.10	0.10	0.26
	**High**	0.52	0.40	0.12	0.01

B. Three groups-clustering (k-means)

		Coefficient			
	**Achievement group**	**Men**	**Women**	**Gap**	**p-value***
**SIMCE**	**Low**	0.00	0.20	-0.20	0.18
	**Medium**	0.25	0.37	-0.11	0.12
	**High**	0.64	0.75	-0.11	0.10
**PSU**	**Low**	0.00	-0.16	0.16	0.29
	**Medium**	0.23	0.11	0.11	0.09
	**High**	0.62	0.54	0.09	0.09

C. Five groups-clustering (k-means)

		Coefficient			
	**Achievement group**	**Men**	**Women**	**Gap**	**p-value***
**SIMCE**	**Low**	0.00	0.25	-0.25	0.22
	**Medium-Low in Math, Medium in Language**	0.44	0.51	-0.07	0.58
	**Medium-Low in Language, Medium in Math**	0.37	0.54	-0.17	0.18
	**Medium-High**	0.75	0.88	-0.13	0.07
	**High**	1.11	1.24	-0.13	0.10
**PSU**	**Low**	0.00	-0.33	0.33	0.07
	**Medium-Low in Math, Medium in Language**	0.15	0.12	0.03	0.83
	**Medium-Low in Language, Medium in Math**	0.16	0.16	0.00	0.94
	**Medium-High**	0.58	0.48	0.10	0.16
	**High**	0.95	0.87	0.08	0.21

Estimates made by controlling for the school's mean 10th grade Mathematics SIMCE test score, attendance rate, Mathematics grades, expectation of students to enter the university, and household fixed effects.

* p-value in the F test to prove the null hypothesis of equality of coefficients.

Table S10. Gender gaps comparison for different levels of previous performance. Estimations based on Model (3) for Language, using alternative student classifications (2)

A. Three groups-classification

		Coefficient			
	**Achievement group**	**Men**	**Women**	**Gap**	**p-value***
**SIMCE**	**Low**	0.00	0.21	-0.21	0.09
	**Medium**	0.32	0.41	-0.09	0.28
	**High**	0.66	0.78	-0.12	0.07
**PSU**	**Low**	0.00	-0.07	0.07	0.61
	**Medium**	0.36	0.22	0.14	0.03
	**High**	0.71	0.63	0.08	0.11

B. Four groups-classification

		Coefficient			
	**Achievement group**	**Men**	**Women**	**Gap**	**p-value***
**SIMCE**	**Low**	0.00	0.13	-0.13	0.20
	**Low in Math, High in Language**	0.21	0.41		0.17
	**Low in Language High in Math**	0.21	0.26	-0.05	0.80
	**High**	0.57	0.68	-0.11	0.05
**PSU**	**Low**	0.00	-0.18	0.18	0.10
	**Low in Math, High in Language**	0.03	0.15	-0.12	0.41
	**Low in Language High in Math**	0.26	0.04	0.22	0.11
	**High**	0.50	0.39	0.11	0.04

Estimates made by controlling for the school's mean 10th grade Mathematics SIMCE test score, attendance rate, Mathematics grades, expectation of students to enter the university, and household fixed effects.

* p-value in the F test to prove the null hypothesis of equality of coefficients.
