# Supplementary material for: Gender gaps in Mathematics and Language: The bias of competitive achievement tests
Source: PLoS One. 2023 Mar 22;18(3):e0283384. doi: 10.1371/journal.pone.0283384 (PMC10032501; doi:10.1371/journal.pone.0283384)
Supplement: S4 Appendix — (PDF) [file pone.0283384.s004.pdf]

S4 Appendix: Additional estimates to study temporal trends that could affect estimations

Students take the tests analyzed at different educational levels; the competitive test is taken in grade 12th and the non-competitive test is taken in grade 10th. To corroborate that the observed differences in performance are due to the characteristics of both tests (competitive and noncompetitive) and not to a temporal trend, we perform a more detailed analysis. In particular, we estimate the same three models from the main text for Language and Mathematics, but instead of using PSU and 10th grade SIMCE scores as outcome variables, we use 10th and 12th grade students' school grades. If the differences observed between the PSU and 10th grade SIMCE tests are due to a temporal trend, we should observe the same trend in the comparison of the 10th and 12th grade students' school grades between men and women.

Gender Gap in Mathematics

Table S11 shows the estimations for the models that predict mathematics school grades in 10th and 12th grade. Note that the variable for women is not significant for any high school grades. Additionally, the magnitude of the coefficients is always positive and higher in 12th grade. Therefore, we find no evidence that men are improving their performance faster than women.

Moreover, we find limited evidence of an interaction effect between gender and previous performance. In both estimations of Model (2), men as well as women have higher grades when they have had higher previous SIMCE test scores. Furthermore, the differences between men and women, both in 10th and 12th grade, are not statistically significant (p-values =0.24 and 0.30, respectively). For Model (3), when comparing the results for men and women according to their achievement group (Table S12), there are only statistically significant gaps in the school grades of 10th grade for the low-medium achievement group, where women have higher grades compared to men. Therefore, differences between the PSU and 10th grade SIMCE tests are not caused by a learning trend where men are improving at a faster rate than women.

Table S11. Estimated Coefficients. Models to predict performance in Mathematic 10th and 12th grade High School grades.

	10th grade Grades			12th grade Grades		
	**Model 1**	**Model 2**	**Model 3**	**Model 1**	**Model 2**	**Model 3**
**Women**	0.015	0.04		0.064	0.092	
**Men * Previous mathematics SIMCE score**[1]		0.147**			0.254***	
**Women * Previous mathematics SIMCE score**[1]		0.102*			0.211**	
**Man * Medium-low achievement**			0.060			0.013
**Men * Medium-high achievement**			0.261			0.114
**Men * High achievement**			0.312***			0.227
**Women * Low achievement**			-0.030			0.096
**Women * Medium- low achievement**			0.258			0.054
**Women * Medium- high achievement**			0.228			0.139
**Women * High achievement**			0.260			0.277
**Previous Mathematics SIMCE score**[1]	0.124**			0.234***		
**Previous Language SIMCE score**[1]	-0.017	-0.015		-0.022	-0.021	
**10th grade mathematics SIMCE school score** [1]	-0.084	-0.086	-0.084	-0.001	0.002	0.118
**Mathematics grades** [2]	0.787***	0.787***	0.806***	0.600***	0.600***	0.667***
**Attendance rate (%)**	0.005	0.005	0.004	0.014	0.014	0.014
Students with university entrance expectation [3]	0.127	0.123	0.121	0.145*	0.138	0.155***
**Constant**	-0.461	-0.507	-0.526	-1.578	-1.572	-1.696
**N**	1136	1136	1136	1136	1136	1136
**Adjusted R^2**	0.657	0.657	0.652	0.603	0.602	0.582

[1] SIMCE and PSU variables were standardized to a distribution with mean equal to zero, and standard deviation equal to 1—standardization made by cohort.
[2] School grades were standardized to a distribution with mean equal to zero, and standard deviation equal to 1—standardization made by cohort and school.
[3] Students with university entrance expectation is a dummy variable. Its value equals 1 when the student in 10th grade expected to attend the university, and 0 otherwise.
* $p<0.05$, ** $p<0.01$, *** $p<0.001$. Estimations include household fixed effects.

Table S12. Gender gap comparison for different levels of previous performance. Estimations based on Model (3) for Mathematics grades.					
		Coefficient			
	Achievement group	Men	Women	Gap	p-value*
10th grade	Low	0.00	-0.03	0.03	0.85
	Medium-Low in Math, Medium in Language	0.06	0.26	-0.2	0.02
	Medium-Low in Language, Medium in Math	0.26	0.23	0.03	0.58
	Medium-High	0.31	0.26	0.05	0.27
12th grade	Low	0.00	0.09	-0.09	0.61
	Medium-Low in Math, Medium in Language	0.01	0.05	-0.04	0.71
	Medium-Low in Language, Medium in Math	0.11	0.14	-0.03	0.72
	Medium-High	0.23	0.28	-0.05	0.39

* p-value in the F test to prove the null hypothesis of equality of coefficients.

Gender Gap in Language

Additionally, Table **S13** shows the results of the models that explain 10th and 12th grade school grades in Language. In both cases, the variable for women has a positive and statistically significant effect. Moreover, the results for model (2) indicate that the effect of the previous SIMCE score on students' school grades in the 10th and 12th grade is not statistically different between men and women (p-value =0.53 and 0.31, respectively).

Finally, Table S14 uses model (3) to compare the coefficients for men and women in each achievement group. We find that, with a 95% confidence level, there are no gender gaps in any of the school grades in the medium-low performance group. For the medium-high achievement group, the gender gap in school grades for 10th grade is not statistically significant, but it is significant for 12th grade. In particular, women in the medium-high group have higher 12th grades when compared to men who are in the same performance group. In contrast, in the high and low performance groups, in 10th grade, there is a gap in favor of women, which is not significant in 12th grade. Thus, it is possible that in the lowest and highest performing groups, men are able to improve their school grades between the 10th and 12th grade at a higher rate than women. We found the opposite effect for the high-performing group at the aggregate level, where women have higher grades in Language in 12th grade compared to men (Model (1) in S13).

In aggregate terms, it seems unlikely that the observed differences between 10th grade SIMCE and PSU scores are explained by a temporal trend in which men or women improve their performance at a different rate.

Table S13. Estimated Coefficients. Models to predict performance in Language High School grades in the 10th y 12th grade.

	10th grade Grades			12th grade Grades		
	Model 1	Model 2	Model 3	Model 1	Model 2	Model 3
**Women**	0.141***	0.128**		0.124***	0.146**	
**Men * Previous mathematics SIMCE score[1]**		0.051			0.084	
**Women * Previous mathematics SIMCE score[1]**		0.076			0.039	
**Men * Medium-low achievement**			0.166			0.191
**Men * Medium-high achievement**			0.277***			0.136
**Men * High achievement**			0.361***			0.311
**Women * Low achievement**			0.382***			0.233
**Women * Medium- low achievement**			0.228			0.206
**Women * Medium- high achievement**			0.396***			0.280
**Women * High achievement**			0.507***			0.396***
**Previous Mathematics SIMCE score[1]**	0.08	0.078		0.164**	0.163**	
**Previous Language SIMCE score[1]**	0.063			0.062		
**10th grade Mathematics SIMCE school score [1]**	-0.043	-0.042	-0.037	-0.153	-0.154	-0.060
**Mathematics grades[2]**	0.679***	0.680***	0.697***	0.677***	0.681***	0.719***
**Attendance rate**	-0.002	-0.002	-0.002	0.007	0.007	0.009
**Students with university entrance expectations[3]**	0.07	0.072	0.083	0.126	0.121	0.163
**Constant**	0.098	0.11	-0.103	-0.877	-0.883	-1.217***
**N**	1064	1064	1064	1064	1064	1064
**Adjusted R^2**	0.574	0.574	0.572	0.577	0.577	0.574

[1] SIMCE and PSU variables were standardized to a distribution with mean equal to zero, and standard deviation equal to 1—standardization made by cohort.
[2] School grades were standardized to a distribution with mean equal to zero, and standard deviation equal to 1—standardization made by cohort and school.
[3] Students with university entrance expectation is a dummy variable. Its value equals 1 when the student in 10th grade expected to attend the university and 0 otherwise.
* p<0.05, ** p<0.01, *** p<0.001. Estimations include household fixed effects.

Table S14. Gender gap comparison for different levels of previous performance. Estimation based on Model (3) for Language grades.

		Coefficient			
	**Achievement group**	**Men**	**Women**	**Gap**	**p-value***
**10th grade**	**Low**	0.00	0.38	-0.38	0.01
	**Medium-Low in Math, Medium in Language**	0.17	0.23	-0.06	0.49
	**Medium-Low in Language, Medium in Math**	0.28	0.40	-0.12	0.08
	**Medium-High**	0.36	0.51	-0.15	0.01
**12th grade**	**Low**	0.00	0.23	-0.23	0.28
	**Medium-Low in Math, Medium in Language**	0.19	0.21	-0.02	0.90
	**Medium-Low in Language, Medium in Math**	0.14	0.28	-0.14	0.04
	**Medium-High**	0.31	0.40	-0.09	0.13

* p-value in the F test to prove the null hypothesis of equality of coefficients.
